# Chemically Modified HKUST-1(Cu) for Gas Adsorption and Separation: Mixed-Metal and Hierarchical Porosity

**DOI:** 10.1021/acsami.4c15059

**Published:** 2024-11-12

**Authors:** Ana Yañez-Aulestia, Víctor M. Trejos, J. Marcos Esparza-Schulz, Ilich A. Ibarra, Elí Sánchez-González

**Affiliations:** † Laboratorio de Fisicoquímica de Superficies, Departamento de Química, Universidad Autónoma Metropolitana-Iztapalapa (UAM-I), C.P. 09310, Ciudad de México, Mexico; ‡ Laboratorio de Fisicoquímica y Reactividad de Superficies (LaFReS), Instituto de Investigaciones en Materiales, Universidad Nacional Autónoma de México, 04510, Ciudad de México, Mexico; § On sabbatical as “Catedra Dr Douglas Hugh Everett” at Departamento de Química, Universidad Autónoma Metropolitana-Iztapalapa, San Rafael Atlixco 186, Col. Leyes de Reforma 1ra Seccion, Iztapalapa, C.P. 09310, Ciudad de México, Mexico

**Keywords:** separation, hierarchical, CO_2_, SO_2_, metal−organic frameworks, transition metals

## Abstract

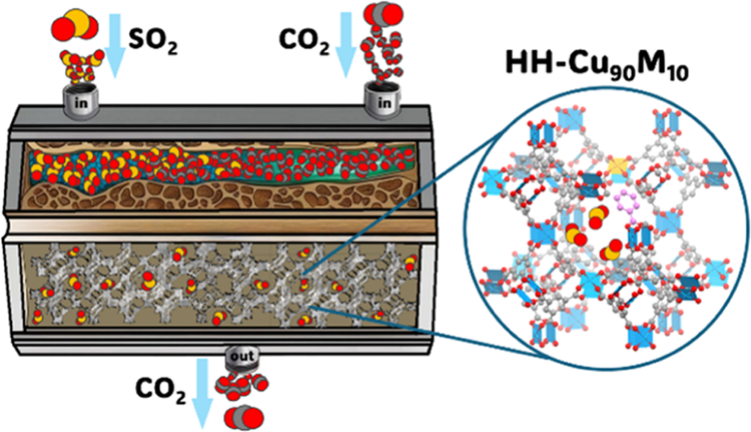

The archetypical metal–organic framework (MOF), HKUST-1, has been systematically modified in both its organic and inorganic building blocks to introduce diversity in the metal centers and create defects within the network, achieving a variety of bimetallic hierarchical structures. These modifications changed the affinity of the MOFs for acid gases. The introduction of bimetallic sites mostly affects CO_2_ adsorption, while the hierarchical structure generates an increase in SO_2_ uptake capacity, allowing better performance in the separation of binary mixtures of these gases near room temperature. Notably, the synthesized HH-Cu_100_ material exhibited an exceptionally high IAST SO_2_/CO_2_ (10:90) selectivity of 3420 at 298 K, outperforming benchmark MOFs with open metal sites.

## 1 Introduction

One of the most studied metal–organic frameworks (MOFs) is HKUST-1 [Cu_3_(BTC)_2_(H_2_O)_3_], first reported by Chui and co-workers.^1^ HKUST-1 contains a characteristic paddlewheel-like secondary building unit (SBU), which is formed by four carboxylate groups coordinated to two Cu^2+^ ions and two solvent molecules to complete the coordination sphere. These solvent molecules are labile coordinated; thus, unsaturated metal sites are generated upon removal. These so-called open metal sites in HKUST-1 are the key features that allowed this MOF to be applied in the adsorption, separation, and catalytic process.^2,3^ Most of its pores are microporous systems contributing to a large surface area, abundant active sites, strong substrate interactions, and effective stabilization of small active species. However, the small size of these micropores limits mass transfer and constrains their potential applications.^4^

Mixed-component metal–organic framework materials incorporate different linkers or metals that play identical structural roles. Many of these mixed-ligand or mixed-metal MOFs exist as solid solutions where the ratios of ligands or metals can be varied or precisely controlled. These materials can be synthesized directly by incorporating multiple metals or ligands during solvothermal synthesis or by applying postsynthetic modifications.^5^ The potential for introducing advanced complexity into MOF materials is significant as the ability to fine-tune or control the ratios of organic and inorganic components within the structure allows for novel adjustments to pore sizes and compositions. This tailoring extends the physicochemical properties of these materials beyond what is achievable with single-component parent MOFs.^6^ One approach to improve the HKUST-1 properties is to mix the metal center, leveraging the synergistic effect of different kinds of metals and their role in adsorption processes. Some reports of the synthesis of bimetallic Ni/Cu-BTC,^7^ Zn/Cu-BTC,^8,9^ V/Cu-BTC,^10^ and Ru/Cu-BTC^11^ by solvothermal approach displayed the same crystalline structure as the monometallic counterpart HKUST-1. Another approach to tackle this challenge involves the development of hierarchically porous MOFs featuring interconnected micro-, meso-, and macropores. These structures are increasing attention due to their multiscale structural regulation, which enhances their potential applications such as storage, catalysis and separation.^12−14^ Hierarchically porous MOFs can be synthesized using either soft or hard templates or by introducing pores through postmodification techniques. Alternatively, they chose a simple method to produce hierarchically porous HKUST-1 using a mixed-ligand strategy to self-assemble simultaneously without changing the crystal structure.^15^ In another approach, hierarchically porous defect HKUST-1 was synthesized using a microwave-assisted method. The dosage of the template reagent cetyltrimethylammonium bromide (CTAB) was adjusted to control defects in the hierarchical pores. This method effectively reduced the diffusion limitations and enhanced the adsorbent-adsorptive interactions.^16^ Wang and collaborators^17^ prepared HKUST-1 with dual pore structures, mesoporous and microporous, using a solvothermal method. The pore sizes were 0.878 and 3.820 nm, respectively.

**Figure 1:**
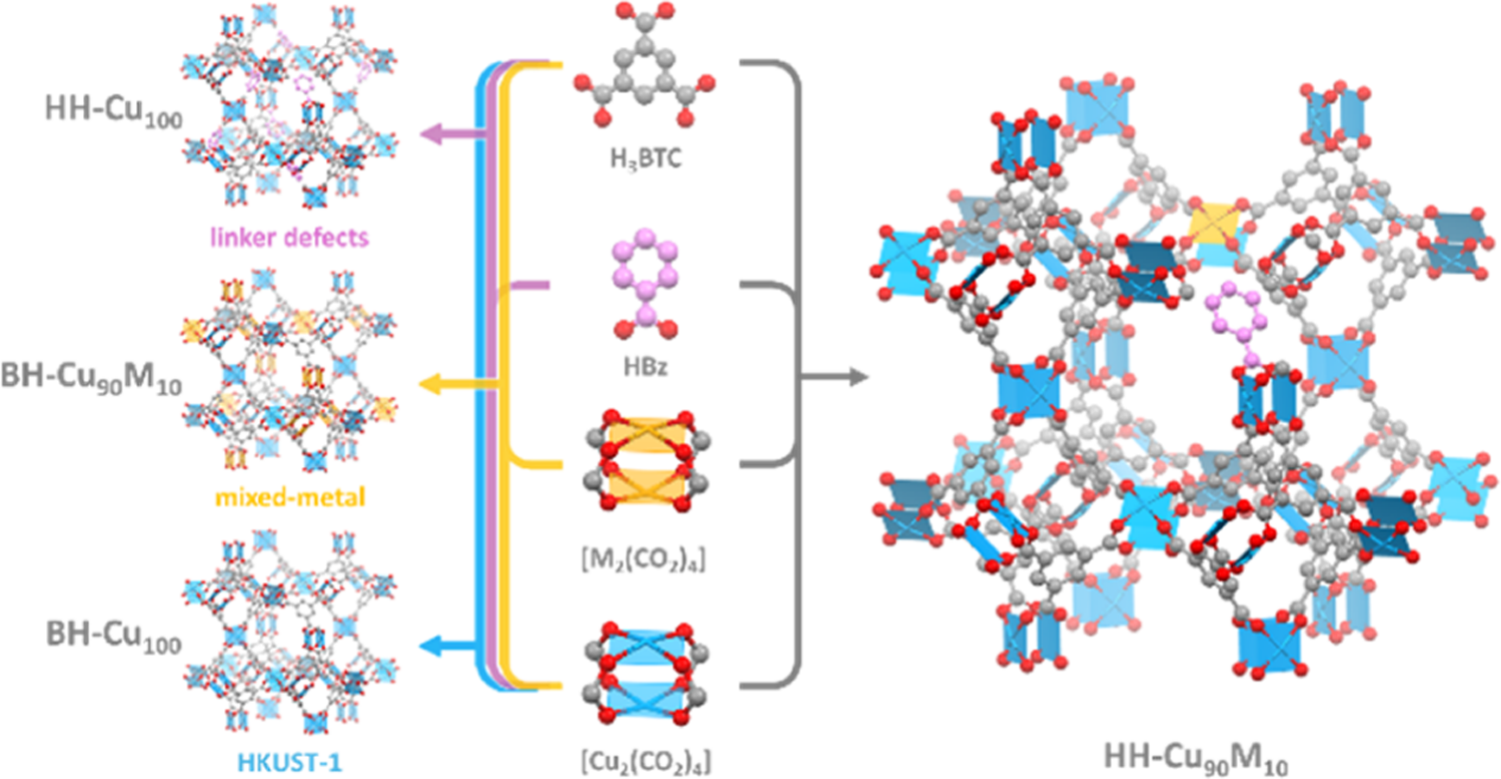
Diversity of structure modifications into the HKUST-1: partial linker substitution to introduce linker defects (HH-Cu_100_); partial metal substitution on the paddlewheel SBU to obtain mixed-metal MOF (BH-Cu_90_M_10_); and both modifications to obtain HH-Cu_90_M_10_ (H_3_BTC: benzene-1,3,5-tricarboxylic acid, HBz: benzoic acid, M: divalent transition metals).

The present investigation focuses on developing bimetallic centers and hierarchically porous HKUST-1 using the solvothermal method by systematically introducing defects with Cu partially substitution by divalent transition metal (Co, Ni, and Rh) and benzoic acid as modulator (Figure 1). The textural properties of these materials were evaluated to assess the impact of these modifications and hierarchization as well as the CO_2_ and SO_2_ adsorption. Finally, their potential use as an adsorbent for acid gas separation, such as SO_2_/CO_2_ separation in flue gas desulfurization.

## 2 Experimental Section

### 2.1 Synthesis of Materials

The HKUST-1, both pristine and modified, were synthesized using the solvothermal method following a methodology described in the literature,^1,18,19^ with some adjustments. The pristine HKUST-1(Cu) was obtained by mixing cupric nitrate hemi pentahydrate (Cu(NO_3_)_2_·2.5H_2_O, 1.5 mmol) dissolved into 5 mL of deionized water, followed by the addition of benzene-1,3,5-tricarboxylic acid (H_3_BTC, 1 mmol) previously dissolved in 5 mL of ethanol. The mixture was stirred in a 20 mL scintillation vial for 2 h at room temperature and then left for 24 h at 393 K. The resulting blue precipitate obtained was washed with ethanol and water 3 times, and an acetone exchange was performed. Finally, the blue precipitate was collected and dried under a vacuum, denoted as BH-Cu_100_. For the first group of materials, HKUST-1 is to substitute 10% mol of Cu with Ni, Co, and Rh. For this purpose, 10% mol of Cu(NO_3_)_2_·2.5H_2_O was replaced with Ni(NO_3_)_2_·6H_2_O, Co(NO_3_)_2_·6H_2_O or [Rh(C_4_H_6_O_4_)]_2_, and the procedure described above was followed to obtain BH-Cu_90_Ni_10_, BH-Cu_90_Co_10,_ and BH-Cu_90_Rh_10_, respectively. Then, the metal ion and ligand were partially substituted in the second group of materials. In this case, two solutions were prepared. The first solution contained 2.06 mmol of Cu(NO_3_)_2_·2.5H_2_O, and 0.22 mmol of the corresponding nitrate (Ni or Co) or rhodium acetate ([Rh(C_4_H_6_O_4_)]_2_) were dissolved in 4 mL of water. The second solution was obtained by mixing 1.9 mmol of H_3_BTC and 0.55 mmol of benzoic acid in 8 mL of DMF:ethanol (*v*/*v* = 1:1). After that, these two solutions were combined in a 20 mL scintillation vial and stirring was continued for another 2 h at room temperature. After stirring, the mixture was heated to 393 K for 24 h for the solvothermal method. After crystallization, the collected precipitate was filtered, dried in air, and washed 3 times with ethanol. Finally, the products were centrifuged and dried under a vacuum overnight. The resulting hierarchically structured HKUST-1 samples were denoted as HH-Cu_90_Ni_10_, HH-Cu_90_Co_10,_ and HH-Cu_90_Rh_10_, respectively. The hierarchically HKUST-1 with 100% of Cu, denoted HH-Cu_100_, was synthesized using the same procedure described for the second group of materials using 2.29 mmol of Cu(NO_3_)_2_·2.5H_2_O.

### 2.2 Characterization

All these samples were initially characterized by powder X-ray diffraction (PXRD). The PXRD patterns were obtained with a D5000 Siemens diffractometer coupled to a Cu anode X-ray tube radiation CuK_α1_ (λ = 1.5406 Å) in the 2θ range from 5 to 50°. Compounds were identified using the Joint Committee Powder Diffraction Standards (JCPDS) database, and cell parameters were obtained by Unicell Software.^20^ After the structural identification, samples were characterized by scanning electron microscopy (SEM) and N_2_ adsorption. The SEM images were obtained by using the secondary electron detector of a JEOL JMS-7600F microscope. In the same microscope, the samples were also analyzed by energy-dispersive X-ray (EDX) to determine their point surface composition. For the N_2_ adsorption–desorption isotherms, the 3P Micro 200 Surface Area and Porosity Analyzer was used. These experiments were performed at 77 K, and all the samples were previously degassed at 383 K for 4 h under a dynamic vacuum. The specific surface area was determined using the Brunauer–Emmett–Teller (BET) method,^21−23^ as implemented in the BETSI Software (BETSI is distributed under the MIT open-source license).^24^ The pore size distributions were calculated using NLDFT kernel fitting aided by the PyGAps software package.^25^

### 2.3 Sorption Experiments

#### 2.3.1 Gas Isotherms

Adsorption isotherms were conducted using 3P Micro 200 Surface Area and Porosity Analyzer equipment in the pressure interval from 0.001 to 1.000 bar at different temperatures: 298 K for N_2_ and 283, 288, 293, and 298 K for CO_2_. The SO_2_ isotherms were recorded at 298 K and up to 1 bar with a dynamic gravimetric gas/vapor sorption analyzer, DVS Vacuum (Surface Measurements Systems Ltd.). Samples were degassed at 383 K for 4 h under vacuum before the measurements. After the sorption tests, the materials were characterized by PXRD, Fourier transform infrared (FTIR), and SEM.

Aditionally, the statistical associating fluid theory for potentials of variable range (SAFT-VR) was tested as an example using the experimental data (CO_2_ isotherms). This theory uses a two-dimensional approach to describe the adsorption of chain fluids (details in Supporting Information).^26,27^

## Results and Discussion

**Figure 2:**
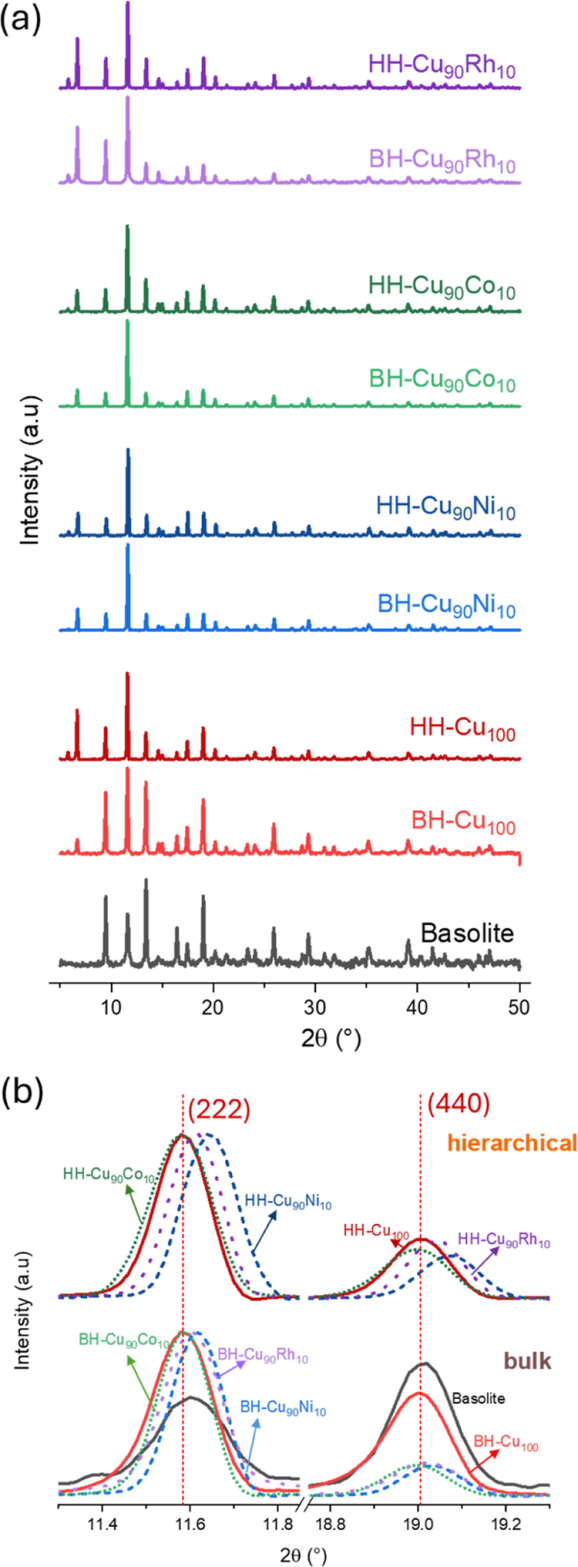
(a) PXRD patterns of HKUST-1 bulk and hierarchical samples; (b) with specific zooms to the (222) and (440) crystal planes. The commercial HKUST-1(Basolite) PXRD pattern is shown for comparison purposes.

The PRXD diffractograms of bulk and modified MOFs results showed one previously reported.^28^ crystal phase corresponding to HKUST-1 (00–065–1028 PDF Card). In all cases, the diffractograms showed a single phase, indicating that the substituted ions and ligands are part of the crystalline phase, and in the respective cases, the bimetallic MOFs were generated (Figure 2a).

In the diffractograms of the first group of compounds, due to the cation’s substitution, different reflection shifts were observed (Figure 2b, bulk). The diffraction peaks shifted to higher angles by substituting cations compared with the BH-Cu_100_ sample. This trend indicated cell contraction or distortion, producing changes in the cell dimensions. The cell contraction is produced by substituting copper (65 pm) for nickel (63 pm) and cobalt (67 pm) ions, considered a five-coordination number, into a square pyramidal structure.^29^ In the case of rhodium ions, previous reports indicated a trend to form the Rh–Rh bond, which generates a higher shrinkage in the cell parameter. When comparing the second group of compounds, hierarchical HKUST-1, the same trend is observed as described above, where the shift to higher angles of the corresponding reflections is related to the decreasing radius of the ions and how they are coordinated (Figure 2b, hierarchical). However, variations in intensity between bulk HKUST-1 and hierarchical HKUST-1 are noticeable. These differences are due to the presence of mixed ligands, which can affect the standard crystallization process, resulting in defect sites within the crystal lattice and thereby changing the intensity of H-HKUST-1.^15^ To obtain the cell parameter, Unitcell Software^20^ was used, and the values calculated are between 26.300 and 26.359, ±0.002 Å (Table 1).

**Table 1:** Parameters of Bulk and Hierarchical HKUST-1 Obtained by the Characterization and CO_2_ Adsorption Experiments

material	a (Å)	*V*_cell_ (Å^3^)	SA_BET_^BETSI^ (m^2^ g^–1^)	*CO*_2uptake_^298K^ (mmol g^-1^)	*Q*_ads_^exp^ (kJ mol^–1^)	*Q*_ads_^cal^ (kJ mol^–1^)
BH-Cu_100_	26.338	18270.3	1654	2.8	24.7	24.8
HH-Cu_100_	26.300	18192.2	1666	1.0	34.5	40.0
BH-Cu_90_Ni_10_	26.340	18272.6	1242	3.3	13.8	19.9
HH-Cu_90_Ni_10_	26.359	18313.4	1723	2.1	32.0	32.7
BH-Cu_90_Co_10_	26.331	18256.3	2585	2.4	30.1	31.3
HH-Cu_90_Co_10_	26.357	18309.7	1892	1.9	10.2	14.9
BH-Cu_90_Rh_10_	26.329	18252.0	873	2.4	26.4	26.7
HH-Cu_90_Rh_10_	26.315	18222.7	1997	3.0	27.0	29.8

**Figure 3:**
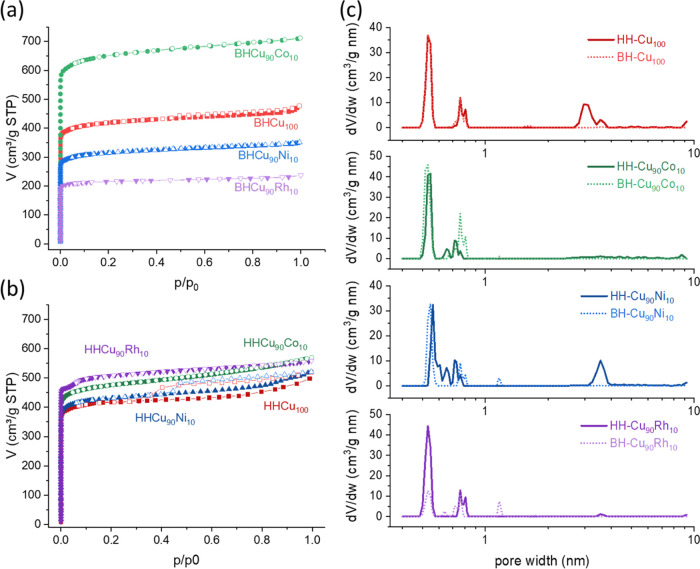
N_2_ adsorption–desorption isotherms of HKUST-1 (a) bulk and (b) hierarchical samples at 77 K and (c) pore size distribution of the bulk (dotted lines) and hierarchical (solid lines) samples.

Figure 3 presents the N_2_ adsorption–desorption isotherms. The bulk HKUST-1 samples showed a typical type-I isotherm, characterized by a sharp increase in uptake at low nitrogen relative pressure, followed by a plateau, indicating its microporous nature (Figure 3a). In contrast, hierarchical HKUST-1 materials exhibited a type IV isotherm as defined by IUPAC^30^ (Figure 3b). At low relative p/p_0_ pressure (0.0 < *p*/*p*_0_ < 0.1), a steep rise in uptake, corresponding to the filling of micropores with nitrogen, was observed in all cases. There is a significant difference in the adsorbed volume for the bulk materials; the volumetric uptake is as follows: BH-Cu_90_Co_10_ > BH-Cu_100_ > BH-Cu_90_Ni_10_ > BH-Cu_90_Rh_10_, no apparent trend related to the ionic radius is observed. The decrease in the volumetric uptake upon adding another metal has been reported for other mixed-metal HKUST-1.^7,9,11,31−34^ Such reduction can be explained by increased defects in the lattice due to metal substitution, as shown by the difference in the intensities in the PXRD patterns (Figure 2). The enhanced volumetric uptake of BH-Cu_90_Co_10_ was unexpected. Still, some enlarged pores can explain this due to the introduced defects, which is reflected in an increase in the contribution of 0.76 and 0.80 nm pores (Figure 3c), almost double compared to the BH-Cu_100_. The hysteresis loop in the hierarchical materials HH-Cu_100_ and HH-Cu_90_Ni_10_ at relatively high pressure (*p*/*p*_0_ > 0.40) suggests capillary condensation of N_2_ in mesopores. The pore size distributions of these two materials show a clear mesopore of about 3.6 nm (Figure 3c); additionally, HH-Cu_100_ exhibits a major mesopore contribution of around 3.0 nm. Despite there being no hysteresis loop for the HH-Cu_90_Co_10_ and HH-Cu_90_Rh_10_ materials, in the pore size distributions, there is a small contribution of mesopores around 3.6 nm (Figure 3c). These findings confirm micropores and mesopores in the HH-Cu_100_ and HH-Cu_90_Ni_10_ samples, while chiefly micropores for the HH-Cu_90_Co_10_ and HH-Cu_90_Rh_10_ samples. Previous reports where defect-engineered MOFs were synthesized via a mixed-ligand approach exhibited pronounced hysteresis loops, indicating hierarchical porosity with mesopores and micropores.^15,16,35^ This is the case for HKUST-1 without any metal addition, where the N_2_ adsorption in the isotherms at low relative pressure region 0.0 < *p*/*p*_0_ < 0.1 for bulk (BH-Cu_100_) and hierarchical (HH-Cu_100_) materials were similar, indicating similar specific surface area calculated by BETSI software^24^ were 1655 and 1660 m^2^ g^–1^, respectively, but the hysteresis loop is the main difference (Table 1 and see Supporting Information). Additionally, the average pore size is smaller in the bulk material than in the hierarchical material, which is explained by the mesoporosity in the latter material.

**Figure 4:**
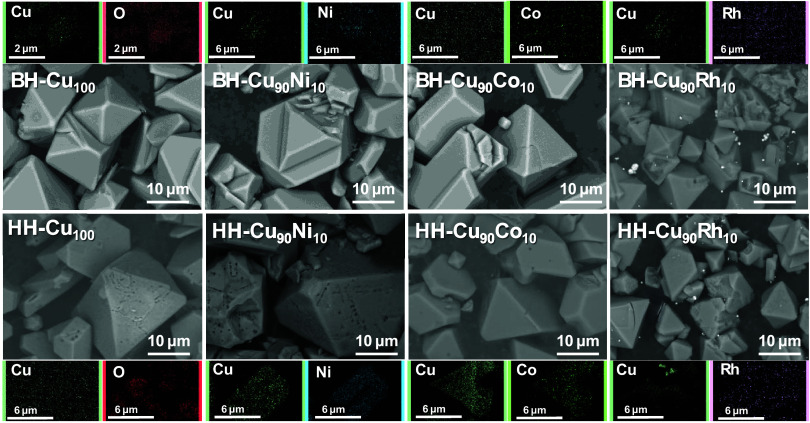
SEM micrographs of HKUST-1 bulk (top) and hierarchical (bottom) samples with their corresponding energy-dispersive X-ray spectroscopy (EDS) elemental mapping images of Cu (green), Ni (blue), Co (light green), Rh (purple), and O (red).

Complementing the characterization, the samples were analyzed by scanning electron microscopy (SEM), where the images of the eight samples showed changes in the HKUST-1 morphology due to the modifications. Figure 4 shows that the conventional B-HKUST-1 sample has a typical single-crystal morphology exhibiting octahedral crystals with slightly circular edges, the ion substitution in the structure favoring the formation of truncated octahedra particles. In contrast, the H-HKUST-1 sample, produced using a mixed-ligand strategy, maintains the overall morphology of the parent HKUST-1. Still, the surfaces are now rugged, featuring irregular needle-like pores of various diameters. This increased surface roughness is likely due to structural defects introduced by substituting benzene-1,3,5-tricarboxylic acid with the benzoic acid ligand. These results are in agreement with the findings reported by Liu.^15^ Additionally, the particle size of hierarchical compounds is approximately 1.5 times larger than that of bulk compounds. In addition to further analyses of the ion’s substitution in the MOF structure, the images obtained by energy-dispersive system (EDS) elemental mapping analysis of each micrograph showed the punctual distribution of Cu, Ni, Co, or Rh ions. It is necessary to mention that copper is homogeneously distributed throughout the particles, with an abundant appearance. The substituent ions are well distributed, and the elemental substitution of the copper ions ranged from 7.4 to 8.4% mol (detailed elemental analysis can be found in Table S3).

**Figure 5:**
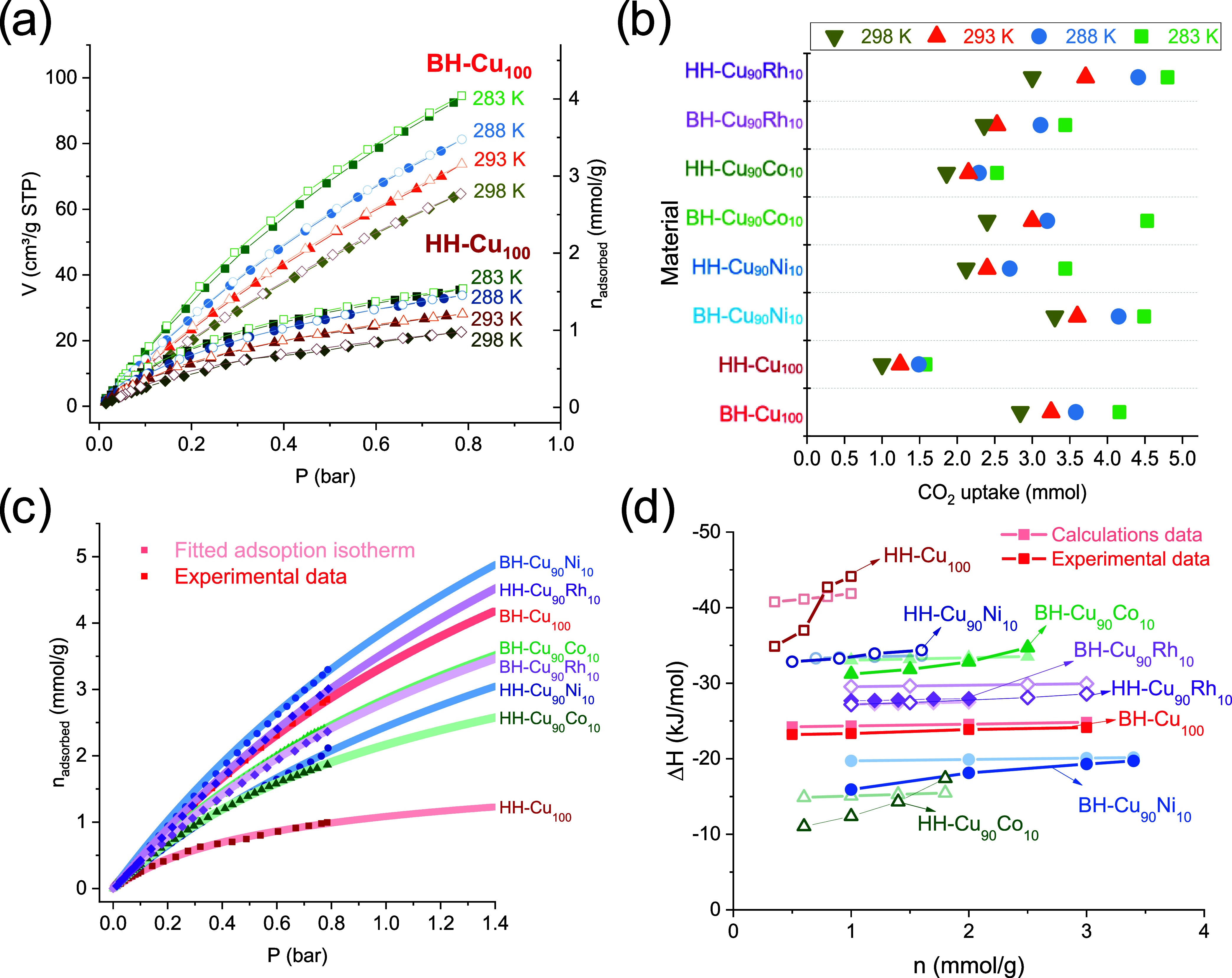
(a) Experimental adsorption–desorption isotherms for BH-Cu_100_ and HH-Cu_100_ materials at 283, 288, 293, and 298 K, (b) CO_2_ uptake of all synthesized materials at 283, 288, 293, and 298 K, (c) fitted CO_2_ adsorption isotherms at 298 K for all synthesized materials, and (d) isosteric heat of adsorption calculated for experimental and fitted CO_2_ adsorption isotherms using Clausius–Clapeyron.

Synthesizing hierarchically porous HKUST-1 by metal and ligand partial substitution aims to cater to the diverse requirements of practical applications, particularly to address high uptake and slow diffusion in gas adsorption (N_2_, CO_2_, and SO_2_). To evaluate the effect of the modifications into HKUST-1, CO_2_ isotherms were performed on the eight materials at four different temperatures. As expected, the increased CO_2_ adsorption capacity with increasing pressure and decreasing temperature indicates physisorption in a microporous solid and the potential suitability for applying the material in pressure or temperature swing adsorption processes.^36^ Likewise, the amount of CO_2_ adsorbed was higher in bulk materials, while in hierarchical compounds, the amount adsorbed was lower. Additionally, CO_2_ adsorption in bulk materials was slightly more temperature-dependent than that in hierarchical materials due to the separation of the isotherms from each other and the variation of the slope of the isotherm as the temperature changes (Figure 5a). This behavior can be explained by the fact that two slightly different Cu^2+^ adsorption sites were present in HKUST-1, mainly attributed to Cu^2+^ present on the external surface and/or related to structural defects and Cu^2+^ present in the inner framework of the MOF. Grajciar and collaborators^37^ reported that the preferential adsorption site is Cu^2+^, which is first occupied by CO_2_ molecules at low loadings due to the strong electrostatic interaction between Cu^2+^ and the quadrupole moment of CO_2_. Still, when the gas concentration was increased, a second adsorption site was detected in the window opening of the small octahedral cage. The secondary adsorption site was attributed to stronger van der Waals interactions since the small cage enables the interaction of a single CO_2_ molecule with multiple surfaces, generating an enhanced adsorption potential. By partially changing the primary adsorption site of CO_2_ by Ni, Co, or Rh, it generates changes in the way the gas molecule is spatially located in the structure, also considering that the gas adsorption amount of substituted sites is different since the adsorption is based on the mass of the corresponding MOF (Figure 5b). In addition to this modification generated by the partial ligand substitution, the secondary adsorption sites also change, tending to decrease due to the partial lack of a bond that forms the octahedron and generates mesoporosity (Figure S1). These experimental data were fed into theoretical calculations applying statistical associating fluid theory for fluids interacting via a variable range pair potential (SAFT-VR).^26,27^ This approach was used to fit the isotherm shape and aid in predicting the data at higher pressures. Figure 5c shows fits to the CO_2_ isotherms at 298 K.

The isosteric heat of adsorption (*Q*_ads_ = −Δ*H*_ads_) for each synthesized material was obtained from the experimental data using the Clausius–Clapeyron approach at four temperatures (Figure 5d) and the fitted model using SAFT-VR. The results explain some behaviors observed in the isotherms, considering that adsorption heats have been considered an indicator of the energetic heterogeneity of an adsorbent in adsorption. (i) In the case of bulk materials, a 10% mol substitution of the metal center generated a change in the CO_2_ adsorption process; the BH-Cu_90_Co_10_ presented the better affinity of CO_2_ and exhibited an enthalpy of −30.1 kJ mol^–1^, followed by BH-Cu_90_Rh_10_, BH-Cu_100_, and BH-Cu_90_Ni_10_ with −26.4, −24.7, and −13.8 kJ mol^–1^ respectively. (ii) For the hierarchical materials, the effect of metal substitution and ligand substitution generates other types of effects; the hierarchical material with the highest affinity to CO_2_ was HH-Cu_100,_ followed by HH-Cu_90_Ni_10_, HH-Cu_90_Rh_10_, and HH-Cu_90_Co_10_ presented −34.5, −32.0, −27.0, and −10.2 kJ mol^–1^, respectively. This trend corresponds well with that observed in the textural characterization, where the hysteresis of the HH-Cu_100_ material is higher than that of HH-Cu_90_Ni_10_, HH-Cu_90_Rh_10_, and HH-Cu_90_Co_10_. (iii) Most bulk materials exhibited lower isosteric heat than hierarchical materials, consistent with the fact that an adsorbent’s energetic heterogeneity is influenced by the distribution of micro- and mesopores of different shapes and sizes and the distribution of adsorption sites of different chemical natures.^38^ This would allow us to explore their selectivity with other gases, especially for HH-Cu_100_ and HH-Cu_90_Ni_10_.

**Figure 6:**
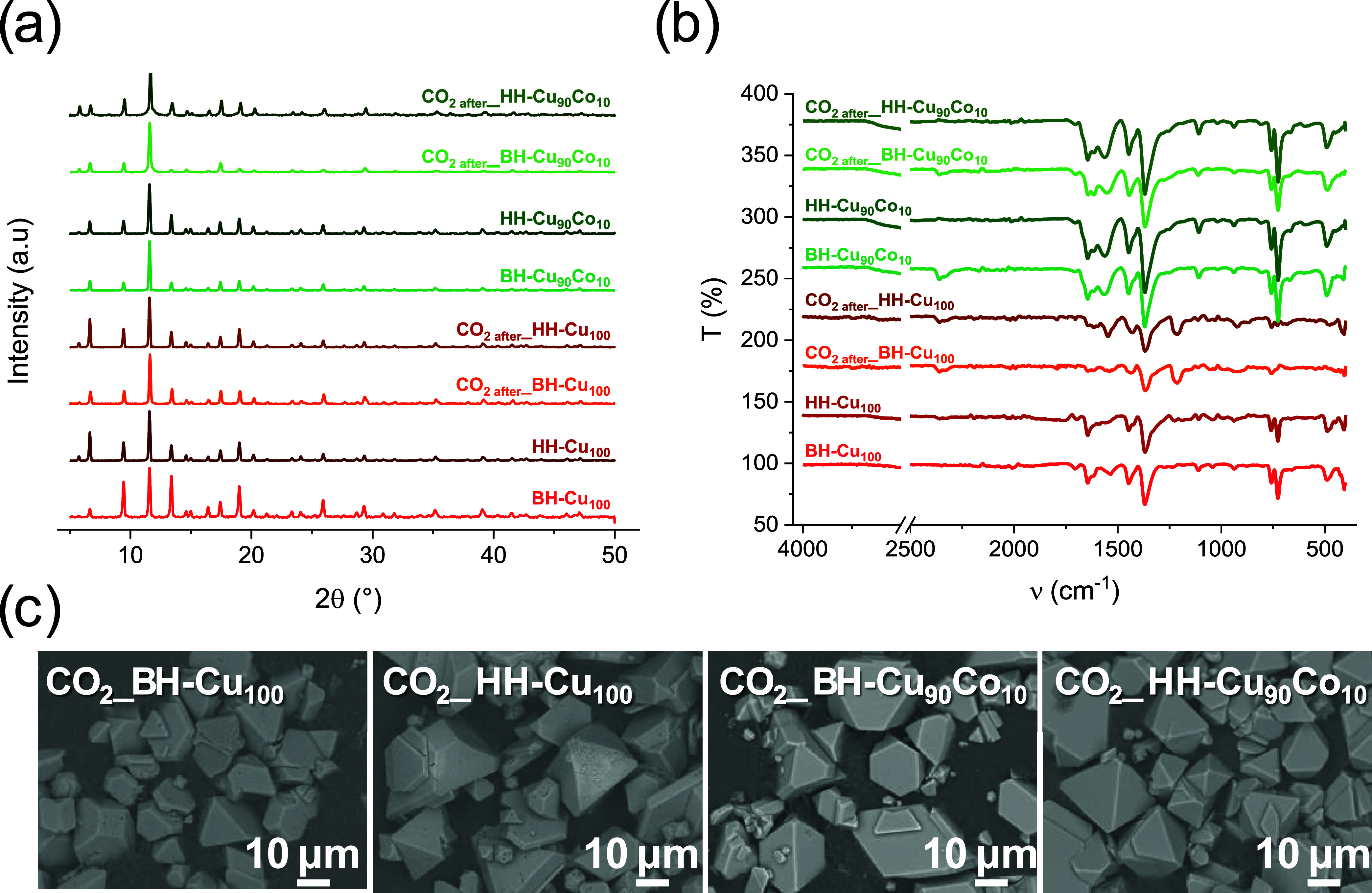
Recharacterization of BH-Cu_100_, HH-Cu_100_, BH-Cu_90_Co_10_, and HH-Cu_90_Co_10_ after CO_2_ capture using (a) PXRD, (b) FTIR-ATR, and (c) SEM.

After the CO_2_ addition experiments, the materials were again characterized by FTIR-ATR, PXRD, and SEM. The results indicate that all the materials are stable and maintain their initial structure (Figure S3). For example, the results obtained for BH-Cu_100_, HH-Cu_100_, BH-Cu_90_Co_10_, and HH-Cu_90_Co_10_ after CO_2_ adsorption are shown (Figure 6). The diffractograms (Figure 6a) fitted the crystalline phase corresponding to HKUST-1 (00–065–1028 PDF Card). IR spectra showed the bands corresponding to the aromatic ring are in 1450, 1106, and 763 cm^–1^ associated with tangential C–C stretching, H–C–C bending, and H–C–C wagging, respectively. The band observed at 490 cm^–1^ represents the only vibration in the IR spectrum that concerns Cu–O bonds. Additionally, the bands at 1645–1446 and 1367 cm^–1^ are associated with two distinct types of carboxylate groups (C–O–O) in antisymmetric and symmetric stretching vibration, respectively (Figure 6b).^39,40^ Then, SEM micrographs were collected to assess any change in the morphology of the samples after the adsorption experiments (Figure 6c). The micrograph showed that the particles did not suffer any deformation; additionally, no formation of any additional phase was observed. These characterizations indicated that the materials remained intact.

**Figure 7:**
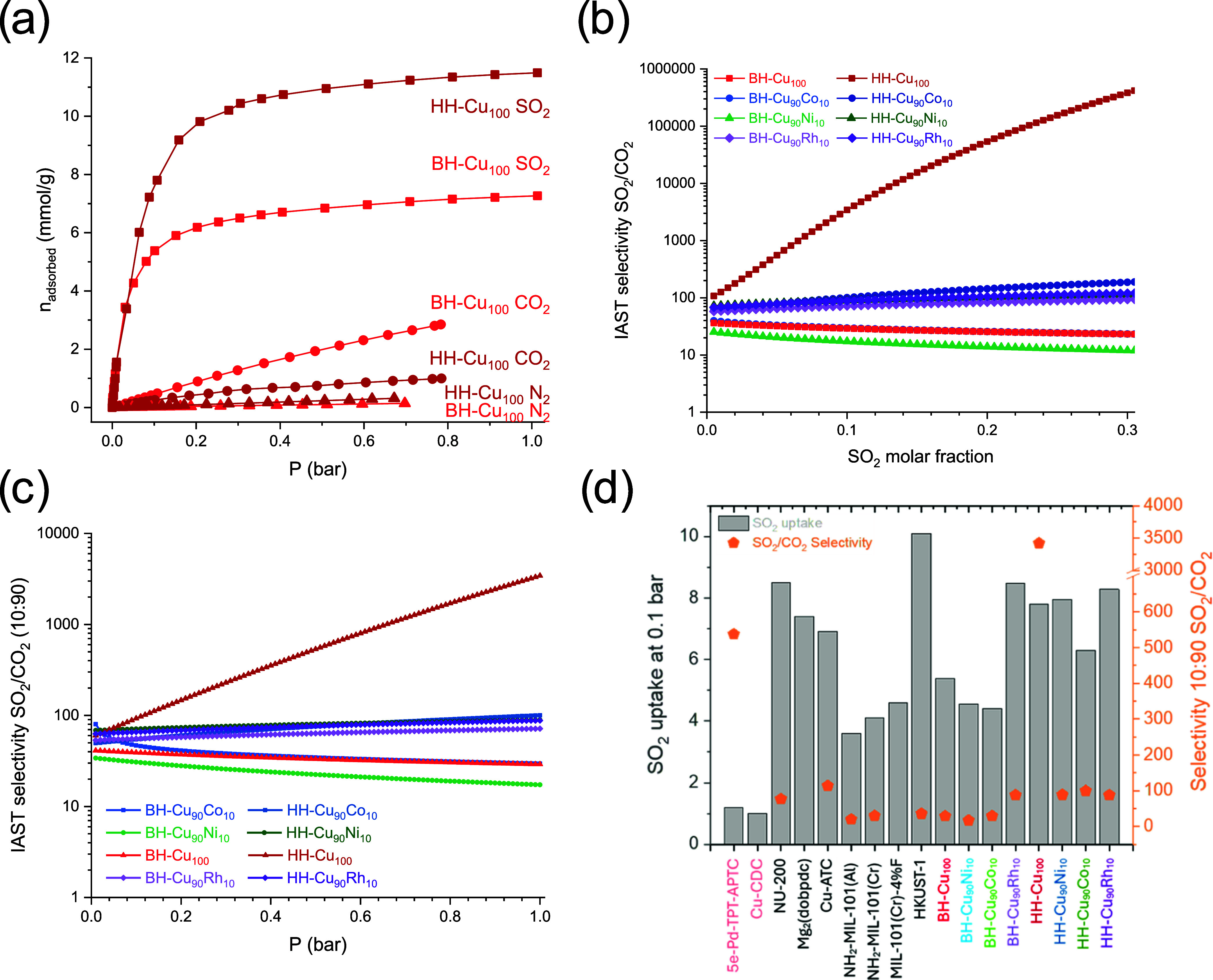
(a) N_2_, CO_2_, and SO_2_ adsorption isotherms comparison at 298 K, (b) IAST selectivity SO_2_/CO_2_ as a function of the SO_2_ molar fraction, (c) IAST selectivity SO_2_/CO_2_ (10:90) at different pressures, and (d) SO_2_ uptake at 0.1 bar and selectivity SO_2_ /CO_2_ (10:90) comparison to metal–organic compounds with open metal sites.

The significant difference in CO_2_ adsorption properties between bulk and hierarchical materials prompts the exploration of sulfur dioxide adsorption, another acid gas with a similar kinetic diameter and physicochemical properties.^41,42^ All materials showed reversible SO_2_ adsorption with type II isotherms (Figure S4), and the framework integrity was corroborated by PXRD (Figure S3). The bulk materials exhibited SO_2_ uptake at 298 K, ranging from 5.9 to 11.7 mmol g^–1^, while the hierarchical materials ranged from 8.6 to 11.5 mmol g^–1^. The hierarchical versions of Cu_100_, Cu_90_Ni_10_, and Cu_90_Co_10_ exhibited an enhanced SO_2_ uptake capacity compared with their bulk counterparts. For example, when comparing the SO_2_ isotherm for BH-Cu_100_ and HH-Cu_100_ (Figure 7a), both materials exhibited a similar uptake until 0.01 bar, with 1.55 and 1.49 mmol g^–1^, respectively. The hierarchical material revealed a higher SO_2_ uptake capacity, 7.43 mmol g^–1^ at 0.1 bar, compared to 5.37 mmol g^–1^ of the bulk BH-Cu_100_ material. After reaching 0.2 bar, both materials became saturated, resulting in a plateau with a slight increase in the uptake, reaching maximum SO_2_ uptake of 7.13 and 11.3 mmol g^–1^ for BH-Cu_100_ and HH-Cu_100_, respectively. The same trend was observed for partially substituted nickel and cobalt materials (Figure S4b,c), obtaining an uptake enhancement of 45, 58 and 73% for HH-Cu_90_Co_10_, HH-Cu_100_, and HH-Cu_90_Ni_10_, respectively (Table 2). For CO_2_ adsorption, bulk materials exhibited a higher uptake; in contrast, for SO_2_ adsorption, the hierarchical versions showed a higher uptake. A plausible hypothesis for these results is that the defects induced by the benzoic linker ease the path to diffuse into the tetrahedral pores of the framework, whose pore-limiting diameter is ∼4 Å, slightly higher than the kinetic diameter of SO_2_ (∼3.6 Å).^43^ Additionally, the inclusion of cobalt and nickel ions reduced the uptake for both bulk and hierarchical materials; this could be associated with the contraction of the metal center upon activation, leading to a more acidic character and thus less compatible with a soft base such as sulfur dioxide. The materials containing rhodium did not significantly differ in the SO_2_ uptake at 298 K; both bulk and hierarchical versions show almost the same profile (Figure S4d), with a slightly higher uptake for the bulk material (11.7 mmol g^–1^) than the hierarchic one (11.5 mmol g^–1^). Compared to the parent’s copper materials, these enhanced uptakes are due to a stronger affinity of sulfur dioxide toward the rhodium sites owed to the π-back bonding stabilization.^44^ The hierarchical materials, in general, present a competitive SO_2_ adsorption at low pressure (between 6.12 and 8.05 mmol g^–1^, for 0.1 bar at 298 K) compared to other MOFs with open metal sites in their structure (Figure 7d), such as NU-200 (8.5 mmol g^–1^),^45^ Mg_2_(dobpdc) (7.4 mmol g^–1^),^46^ Cu-ATC (6.9 mmol g^–1^),^47^ MFM-170 (6.5 mmol g^–1^),^48^ MIL-101(Cr)-4%F (4.6 mmol g^–1^),^49^ NH_2_-MIL-101(Al) (3.6 mmol g^–1^),^50^ NH_2_-MIL-101(Cr) (4.1 mmol g^–1^ at 293 K), and HKUST-1 (10.1 mmol g^–1^ at 293 K).^51^

**Table 2:** Summary of SO_2_ Uptakes, CO_2_ Uptake, and IAST Selectivities for the Synthesized Materials at 298 K

	SO_2_ uptake [mmol g^–1^]				CO_2_ uptake^a^ [mmol g^–1^]	IAST selectivity SO_2_/CO_2_ (10:90)	
material	0.01 bar	0.05 bar	0.1 bar	1 bar	1 bar	1 bar	0.1 bar
BH-Cu_100_	1.55	4.22	5.37	7.13	3.42	29.0	39.1
HH-Cu_100_	1.49	5.15	7.43	11.3	1.08	3420	92.6
BH-Cu_90_Co_10_	1.34	3.46	4.40	5.86	2.93	29.5	46.9
HH-Cu_90_Co_10_	1.60	4.66	6.12	8.63	2.15	100	54.7
BH-Cu_90_Ni_10_	1.33	3.57	4.53	6.27	4.02	17.3	30.7
HH-Cu_90_Ni_10_	1.96	5.80	7.70	10.9	2.44	89.2	71.1
BH-Cu_90_Rh_10_	1.87	5.98	8.24	11.7	2.81	71.7	55.6
HH-Cu_90_Rh_10_	1.85	5.87	8.05	11.5	2.44	88.1	65.7

a Values obtained from extrapolating the Langmuir fit of experimental isotherms.

Flue gas desulfurization is an industrially relevant separation process that removes sulfur dioxide from flue gas mixtures, usually containing CO_2_, N_2_, or CH_4_.^52^ The SO_2_ separation from nitrogen and methane is fairly accessible since both inert gases are less polarizable and do not compete for preferential adsorption sites (Lewis acid, Lewis base or hydrogen bond donors); conversely to the separation from CO_2_ where both gases compete from the same adsorption sites. The presented copper-based MOFs exhibit good adsorbent properties toward acidic gases: high SO_2_ adsorption capacity, reversible adsorption with mild regeneration conditions, and physisorption for both SO_2_ and CO_2_. When the adsorption isotherms of SO_2_, CO_2_, and N_2_ are compared at 298 K (Figure 7a), it is evident that nitrogen does not compete for the adsorption sites at this temperature. Ideal adsorbed solution theory (IAST) was used to estimate the SO_2_/CO_2_ separation selectivity from the experimental monocomponent adsorption isotherms at 298 K (Figure 7b, details in Supporting Information).^53^ The evaluated SO_2_ molar fraction for the binary mixture was in the low range (from 0.005 to 0.3), obtaining a better performance for the hierarchical samples for all of the materials. The selectivity values for the bulk samples ranged from 20 to 93, with the following trend: BH-Cu_90_Rh_10_ > BH-Cu_90_Co_10_ > BH-Cu_100_ > BH-Cu_90_Rh_10_. For the hierarchical samples containing Co, Ni, and Rh, selectivities obtained were above 60, achieving a better performance for the HH-Cu_90_Co_10_ with a value of 188 at a 0.3 SO_2_ molar fraction. The HH-Cu_100_ material exhibited an exceptionally high IAST SO_2_/CO_2_ selectivity of 3420 (10:90 at 1 bar), outperforming benchmark materials such as CPL-1-NH_2_ (485),^54^ Mg-gallate (321),^52^ SIFSIX-Cu-TPA (191),^55^ DMOF-TM (169),^56^ MFM-305 (160),^57^ Co-gallate (143),^52^ MFM-520 (125),^58^ Cu-ATC (114),^47^ SNFSIX-Cu-TPA (88),^55^ SIFSIX-1-Cu-i(87),^59^ NU-200 (77),^45^ and HKUST-1 (36).^51^ Despite the good selectivity of HH-Cu_100_ at 1 bar of SO_2_, the selectivity drops to 92.6 at 0.1 bar (Figure 7c) but is still higher than those of other copper-based MOFs containing open metal sites: NU-200 (90),^45^ Cu-ATC (87),^47^ and MFM-170 (26).^48^ The enhanced selectivity obtained for the hierarchical samples is due to the introduced defects on the structure; that presence slightly increases the pore space, leaving a free path for the SO_2_ molecules to diffuse into, thus leading to higher SO_2_ uptakes. The “extra” pore space reduces interactions between CO_2_ molecules, leading to lower CO_2_ uptake for hierarchical materials than bulk ones. These trademarks stem from the defect generator employed in the synthesis of copper-based MOFs (benzoic acid), leading to higher SO_2_/CO_2_ selectivities. Such drastic effect of the modulator has been reported only for zirconium clusters in the Zr-TPA-FA using formic acid as hemilabile capping agents leading to a dynamic SO_2_ chemisorption;^60^ and DUT-67-HCl using HCl to remove a capping agent (formic acid), leading to free HO-/H_2_O and additional SO_2_ adsorption sites.^61^

## Conclusions

In this work, eight modified HKUST-1 materials with mixed metal and partial ligand substitution were synthesized, characterized, and evaluated for CO_2_ and SO_2_ adsorption and selectivity. The ligand substitution generated defects in the framework, leading to hierarchical porosity confirmed by the N_2_ adsorption isotherms at 77 K. The partial substitution of Cu by Ni, Co, and Rh in the metal centers did not affect the overall framework structure. This metal substitution changed the CO_2_ adsorption capacity and affinity, revealing a clear relationship between the CO_2_ uptake and the metal acidity Ni > Cu > Co. The hierarchical materials consistently exhibited a lower CO_2_ capture and temperature dependence. The HH-Cu_100_ and HH-Cu_90_Co_10_ displayed high energetic heterogeneity due to the variety of micro- and mesopores and the distribution of adsorption sites with diverse chemical properties, reflected in the CO_2_ adsorption enthalpy values. All materials presented a reversible SO_2_ adsorption at 298 K, where the hierarchical materials outperformed the bulk materials. The gas absorption results allowed us to evaluate these materials in the potential separation of binary mixtures of SO_2_ and CO_2_ near room temperature. The HH-Cu_100_ exhibited an exceptionally high IAST SO_2_/CO_2_ selectivity (3420), outperforming other MOFs with open metal sites at 298 K for a 0.1 SO_2_ molar fraction. No significant difference was observed between BH-Cu_90_Rh_10_ and HH-Cu_90_Rh_10_ in terms of SO_2_ capture and SO_2_/CO_2_ selectivity. This research shed light on the potential of introducing lattice defects as a strategy in materials design to improve the separation performance for acid gases.

## Supporting Information Available

Theoretical calculations for the SAFT-VR model; BET surface area calculations; EDS elemental analyses; CO_2_ and SO_2_ isotherms; IAST selectivity; and additional characterization (PDF)

## References

[ref1] Chui S. S.-Y.; Lo S. M.-F.; Charmant J. P. H.; Orpen A. G.; Williams I. D. A Chemically Functionalizable Nanoporous Material [Cu_3_(TMA)_2_(H_2_O)_3_]_n_. Science 1999, 283 (5405), 1148–1150. 10.1126/science.283.5405.1148. 10024237

[ref2] Shekhah O.; Liu J.; Fischer R. A.; Wöll C. MOF Thin Films: Existing and Future Applications. Chem. Soc. Rev. 2011, 40 (2), 1081–1106. 10.1039/c0cs00147c. 21225034

[ref3] Tranchemontagne D. J.; Hunt J. R.; Yaghi O. M. Room Temperature Synthesis of Metal-Organic Frameworks: MOF-5, MOF-74, MOF-177, MOF-199, and IRMOF-0. Tetrahedron 2008, 64 (36), 8553–8557. 10.1016/j.tet.2008.06.036.

[ref4] Xiong Q.; Chen Y.; Yang D.; Wang K.; Wang Y.; Yang J.; Li L.; Li J. Constructing Strategies for Hierarchically Porous MOFs with Different Pore Sizes and Applications in Adsorption and Catalysis. Mater. Chem. Front. 2022, 6, 2944–2967. 10.1039/D2QM00557C.

[ref5] Hou H.; Li G.; Li L.; Zhu Y.; Meng X.; Fan Y. Synthesis, Crystal Structures, and Magnetic Properties of Three Novel Ferrocenecarboxylato-Bridged Lanthanide Dimers. Inorg. Chem. 2003, 42 (2), 428–435. 10.1021/ic025753w. 12693224

[ref6] Tian F.; Qiao C.; Zheng R.; Ru Q.; Sun X.; Zhang Y.; Meng C. Synthesis of Bimetallic-Organic Framework Cu/Co-BTC and the Improved Performance of Thiophene Adsorption. RSC Adv. 2019, 9 (27), 15642–15647. 10.1039/C9RA02372K. 35514848 PMC9064320

[ref7] Hu J.; Yu H.; Dai W.; Yan X.; Hu X.; Huang H. Enhanced Adsorptive Removal of Hazardous Anionic Dye “Congo Red” by a Ni/Cu Mixed-Component Metal–Organic Porous Material. RSC Adv. 2014, 4 (66), 35124–35130. 10.1039/C4RA05772D.

[ref8] Wang T.; Li X.; Dai W.; Fang Y.; Huang H. Enhanced Adsorption of Dibenzothiophene with Zinc/Copper-Based Metal–Organic Frameworks. J. Mater. Chem. A 2015, 3 (42), 21044–21050. 10.1039/C5TA05204A.

[ref9] Gul-E-Noor F.; Jee B.; Mendt M.; Himsl D.; Pöppl A.; Hartmann M.; Haase J.; Krautscheid H.; Bertmer M. Formation of Mixed Metal Cu_3–X_Zn_x_(Btc)_2_ Frameworks with Different Zinc Contents: Incorporation of Zn^2+^ into the Metal–Organic Framework Structure as Studied by Solid-State NMR. J. Phys. Chem. C 2012, 116 (39), 20866–20873. 10.1021/jp3054857.

[ref10] Zhao G.; Liu Q.; Tian N.; Yu L.; Dai W. Highly Efficient Benzothiophene Capture with a Metal-Modified Copper–1,3,5-Benzenetricarboxylic Acid Adsorbent. Energy Fuels 2018, 32 (6), 6763–6769. 10.1021/acs.energyfuels.8b01223.

[ref11] Gotthardt M. A.; Schoch R.; Wolf S.; Bauer M.; Kleist W. Synthesis and Characterization of Bimetallic Metal-Organic Framework Cu-Ru-BTC with HKUST-1 Structure. Dalton Trans. 2015, 44 (5), 2052–2056. 10.1039/C4DT02491E. 25518915

[ref12] Duan C.; Liang K.; Zhang Z.; Li J.; Chen T.; Lv D.; Li L.; Kang L.; Wang K.; Hu H.; Xi H. Recent Advances in the Synthesis of Nanoscale Hierarchically Porous Metal–Organic Frameworks. Nano Mater. Sci. 2022, 4 (4), 351–365. 10.1016/j.nanoms.2021.12.003.

[ref13] Zhang W.; Taheri-Ledari R.; Saeidirad M.; Qazi F. S.; Kashtiaray A.; Ganjali F.; Tian Y.; Maleki A. Regulation of Porosity in MOFs: A Review on Tunable Scaffolds and Related Effects and Advances in Different Applications. J. Environ. Chem. Eng. 2022, 10 (6), 108836 10.1016/j.jece.2022.108836.

[ref14] Jiang L.; Zhou H.; Yang H.; Sun N.; Huang Z.; Pang H. Applications of Hierarchical Metal–Organic Frameworks and Their Derivatives in Electrochemical Energy Storage and Conversion. J. Energy Storage 2022, 55, 105354 10.1016/j.est.2022.105354.

[ref15] Liu B.; Li Y.; Oh S. C.; Fang Y.; Xi H. Fabrication of a Hierarchically Structured HKUST-1 by a Mixed-Ligand Approach. RSC Adv. 2016, 6 (66), 61006–61012. 10.1039/C6RA11917D.

[ref16] Qiu S.; Du J.; Xiao Y.; Zhao Q.; He G. Hierarchical Porous HKUST-1 Fabricated by Microwave-Assisted Synthesis with CTAB for Enhanced Adsorptive Removal of Benzothiophene from Fuel. Sep. Purif. Technol. 2021, 271, 118868 10.1016/j.seppur.2021.118868.

[ref17] Ma X.; Wang L.; Wang H.; Deng J.; Song Y.; Li Q.; Li X.; Dietrich A. M. Insights into Metal-Organic Frameworks HKUST-1 Adsorption Performance for Natural Organic Matter Removal from Aqueous Solution. J. Hazard. Mater. 2022, 424, 126918 10.1016/j.jhazmat.2021.126918. 34775305

[ref18] Vishnyakov A.; Ravikovitch P. I.; Neimark A. V.; Bülow M.; Wang Q. M. Nanopore Structure and Sorption Properties of Cu-BTC Metal-Organic Framework. Nano Lett. 2003, 3 (6), 713–718. 10.1021/nl0341281.

[ref19] Peedikakkal A. M. P.; Aljundi I. H. Mixed-Metal Cu-BTC Metal-Organic Frameworks as a Strong Adsorbent for Molecular Hydrogen at Low Temperatures. ACS Omega 2020, 5 (44), 28493–28499. 10.1021/acsomega.0c02810. 33195899 PMC7658931

[ref20] Holland T. J. B.; Redfern S. A. T. Unit Cell Refinement from Powder Diffraction Data : The Use of Regression Diagnostics. Mineral Mag. 1997, 61 (6), 65–77. 10.1180/minmag.1997.061.404.07.

[ref21] Brunauer S. The Adsorption o Gases and Vapors; Princeton University Press, 1943.

[ref22] Fagerlund G. Determination of Specific Surface by the BET Method. Matér. Constr. 1973, 6 (3), 239–245. 10.1007/BF02479039.

[ref23] Brunauer S.; Emmett P. H.; Teller E. Adsorption of Gases in Multimolecular Layers. J. Am. Chem. Soc. 1938, 60 (2), 309–319. 10.1021/ja01269a023.

[ref24] Osterrieth J. W. M.; Rampersad J.; Madden D.; Rampal N.; Skoric L.; Connolly B.; Allendorf M. D.; Stavila V.; Snider J. L.; Ameloot R.; Marreiros J.; Ania C.; Azevedo D.; Vilarrasa-Garcia E.; Santos B. F.; Bu X. H.; Chang Z.; Bunzen H.; Champness N. R.; Griffin S. L.; Chen B.; Lin R. B.; Coasne B.; Cohen S.; Moreton J. C.; Colón Y. J.; Chen L.; Clowes R.; Coudert F. X.; Cui Y.; Hou B.; D’Alessandro D. M.; Doheny P. W.; Dincă M.; Sun C.; Doonan C.; Huxley M. T.; Evans J. D.; Falcaro P.; Ricco R.; Farha O.; Idrees K. B.; Islamoglu T.; Feng P.; Yang H.; Forgan R. S.; Bara D.; Furukawa S.; Sanchez E.; Gascon J.; Telalović S.; Ghosh S. K.; Mukherjee S.; Hill M. R.; Sadiq M. M.; Horcajada P.; Salcedo-Abraira P.; Kaneko K.; Kukobat R.; Kenvin J.; Keskin S.; Kitagawa S.; Otake K. ichi.; Lively R. P.; DeWitt S. J. A.; Llewellyn P.; Lotsch B. V.; Emmerling S. T.; Pütz A. M.; Martí-Gastaldo C.; Padial N. M.; García-Martínez J.; Linares N.; Maspoch D.; del Pino J. A. S.; Moghadam P.; Oktavian R.; Morris R. E.; Wheatley P. S.; Navarro J.; Petit C.; Danaci D.; Rosseinsky M. J.; Katsoulidis A. P.; Schröder M.; Han X.; Yang S.; Serre C.; Mouchaham G.; Sholl D. S.; Thyagarajan R.; Siderius D.; Snurr R. Q.; Goncalves R. B.; Telfer S.; Lee S. J.; Ting V. P.; Rowlandson J. L.; Uemura T.; Iiyuka T.; van der Veen M. A.; Rega D.; Van Speybroeck V.; Rogge S. M. J.; Lamaire A.; Walton K. S.; Bingel L. W.; Wuttke S.; Andreo J.; Yaghi O.; Zhang B.; Yavuz C. T.; Nguyen T. S.; Zamora F.; Montoro C.; Zhou H.; Kirchon A.; Fairen-Jimenez D. How Reproducible Are Surface Areas Calculated from the BET Equation?. Adv. Mater. 2022, 34 (27), 2201502 10.1002/adma.202201502. 35603497

[ref25] Iacomi P.; Llewellyn P. L. PyGAPS: A Python-Based Framework for Adsorption Isotherm Processing and Material Characterisation. Adsorption 2019, 25 (8), 1533–1542. 10.1007/s10450-019-00168-5.

[ref26] Gil-Villegas A.; Galindo A.; Whitehead P. J.; Mills S. J.; Jackson G.; Burgess A. N. Statistical Associating Fluid Theory for Chain Molecules with Attractive Potentials of Variable Range. J. Chem. Phys. 1997, 106 (10), 4168–4186. 10.1063/1.473101.

[ref27] Martinez A.; Castro M.; McCabe C.; Gil-Villegas A. Predicting Adsorption Isotherms Using a Two-Dimensional Statistical Associating Fluid Theory. J. Chem. Phys. 2007, 126 (7), 074707 10.1063/1.2483505. 17328627

[ref28] Wong-Ng W.; Kaduk J. A.; Siderius D. L.; Allen A. L.; Espinal L.; Boyerinas B. M.; Levin I.; Suchomel M. R.; Ilavsky J.; Li L.; Williamson I.; Cockayne E.; Wu H. Reference Diffraction Patterns, Microstructure, and Pore-Size Distribution for the Copper (II) Benzene-1,3,5-Tricarboxylate Metal Organic Framework (Cu-BTC) Compounds. Powder Diffr. 2015, 30 (1), 2–13. 10.1017/S0885715614001195.

[ref29] Shannon R. D. Revised Effective Ionic Radii and Systematic Studies of Interatomic Distances in Halides and Chalcogenides. Acta Crystallogr., Sect. A 1976, 32 (5), 751–767. 10.1107/S0567739476001551.

[ref30] Lowell S.; Shields J. E.; Thomas M. A.; Thommes M. Characterization of Porous Solids and Powders: Surface Area, Pore Size and Density, Particle Technology Series; Springer: Netherlands, 2012.

[ref31] Lee S.-K.; Hong D.-Y.; Jeong M.-G.; Yoon J. W.; Bae J.; Kim Y. D.; Chang J.-S.; Hwang Y. K. Trimetallic Copper Trimesate with Isomorphously Substituted Mo(VI) and Its Catalytic Properties. Microporous Mesoporous Mater. 2017, 253, 223–232. 10.1016/j.micromeso.2017.07.007.

[ref32] Guo P.; Froese C.; Fu Q.; Chen Y.-T.; Peng B.; Kleist W.; Fischer R. A.; Muhler M.; Wang Y. CuPd Mixed-Metal HKUST-1 as a Catalyst for Aerobic Alcohol Oxidation. J. Phys. Chem. C 2018, 122 (37), 21433–21440. 10.1021/acs.jpcc.8b05882.

[ref33] Gallis D. F. S.; Parkes M. V.; Greathouse J. A.; Zhang X.; Nenoff T. M. Enhanced O _2_ Selectivity versus N _2_ by Partial Metal Substitution in Cu-BTC. Chem. Mater. 2015, 27 (6), 2018–2025. 10.1021/cm5042293.

[ref34] Bitzer J.; Otterbach S.; Thangavel K.; Kultaeva A.; Schmid R.; Pöppl A.; Kleist W. Experimental Evidence for the Incorporation of Two Metals at Equivalent Lattice Positions in Mixed-Metal Metal–Organic Frameworks. Chem. - Eur. J. 2020, 26 (25), 5667–5675. 10.1002/chem.201905596. 31860147 PMC7317703

[ref35] Qiu L. G.; Xu T.; Li Z. Q.; Wang W.; Wu Y.; Jiang X.; Tian X. Y.; Zhang L. De. Hierarchically Micro- and Mesoporous Metal-Organic Frameworks with Tunable Porosity. Angew. Chem., Int. Ed. 2008, 47 (49), 9487–9491. 10.1002/anie.200803640. 18972472

[ref36] D’Alessandro D. M.; Smit B.; Long J. R. Carbon Dioxide Capture: Prospects for New Materials. Angew. Chem., Int. Ed. 2010, 49 (35), 6058–6082. 10.1002/anie.201000431. 20652916

[ref37] Grajciar L.; Wiersum A. D.; Llewellyn P. L.; Chang J. S.; Nachtigall P. Understanding CO_2_ Adsorption in CuBTC MOF: Comparing Combined DFT-Ab Initio Calculations with Microcalorimetry Experiments. J. Phys. Chem. C 2011, 115 (36), 17925–17933. 10.1021/jp206002d.

[ref38] Sircar S.; Rao M. B. Surfaces of Nanoparticles and Porous Materials; Schwarz J.; Contescu C., Eds.; CRC press: New York, 1999; Vol. 78.

[ref39] Dhumal N. R.; Singh M. P.; Anderson J. A.; Kiefer J.; Kim H. J. Molecular Interactions of a Cu-Based Metal-Organic Framework with a Confined Imidazolium-Based Ionic Liquid: A Combined Density Functional Theory and Experimental Vibrational Spectroscopy Study. J. Phys. Chem. C 2016, 120 (6), 3295–3304. 10.1021/acs.jpcc.5b10123.

[ref40] Gentile F. S.; Pannico M.; Causà M.; Mensitieri G.; Di Palma G.; Scherillo G.; Musto P. Metal Defects in HKUST-1 MOF Revealed by Vibrational Spectroscopy: A Combined Quantum Mechanical and Experimental Study. J. Mater. Chem. A 2020, 8 (21), 10796–10812. 10.1039/D0TA01760D.

[ref41] Baksh M. S. A.; Yang R. T. Unique Adsorption Properties and Potential Energy Profiles of Microporous Pillared Clays. AIChE J. 1992, 38 (9), 1357–1368. 10.1002/aic.690380906.

[ref42] Li J.-R.; Kuppler R. J.; Zhou H.-C. Selective Gas Adsorption and Separation in Metal–Organic Frameworks. Chem. Soc. Rev. 2009, 38 (5), 1477–1504. 10.1039/b802426j. 19384449

[ref43] Jia J.; Bhatt P. M.; Tavares S. R.; Abou-Hamad E.; Belmabkhout Y.; Jiang H.; Mallick A.; Parvatkar P. T.; Maurin G.; Eddaoudi M. Porous Organic Polymers for Efficient and Selective SO_2_ Capture from CO_2_ -rich Flue Gas. Angew. Chem., Int. Ed. 2024, 63 (26), e202318844 10.1002/anie.202318844. 38785268

[ref44] Hrdina R. Dirhodium(II,II) Paddlewheel Complexes. Eur. J. Inorg. Chem. 2021, 2021 (6), 501–528. 10.1002/ejic.202000955.

[ref45] Gong W.; Xie Y.; Pham T. D.; Shetty S.; Son F. A.; Idrees K. B.; Chen Z.; Xie H.; Liu Y.; Snurr R. Q.; Chen B.; Alameddine B.; Cui Y.; Farha O. K. Creating Optimal Pockets in a Clathrochelate-Based Metal–Organic Framework for Gas Adsorption and Separation: Experimental and Computational Studies. J. Am. Chem. Soc. 2022, 144 (8), 3737–3745. 10.1021/jacs.2c00011. 35179374

[ref46] Martínez-Ahumada E.; Kim D. w.; Wahiduzzaman M.; Carmona-Monroy P.; López-Olvera A.; Williams D. R.; Martis V.; Lara-García H. A.; López-Morales S.; Solis-Ibarra D.; Maurin G.; Ibarra I. A.; Hong C. S. Capture and Detection of SO _2_ Using a Chemically Stable Mg(II) – MOF. J. Mater. Chem. A 2022, 10 (36), 18636–18643. 10.1039/D2TA04842F.

[ref47] Zhu Z.; Wu K.; Liu X.; Zhang P.; Chen S.; Chen J.; Deng Q.; Zeng Z.; Deng S.; Wang J. Dense Open Metal Sites in a Microporous Metal–Organic Framework for Deep Desulfurization with Record-high Sulfur Dioxide Storage Density. AIChE J. 2022, 68 (9), e17811 10.1002/aic.17811.

[ref48] Smith G. L.; Eyley J. E.; Han X.; Zhang X.; Li J.; Jacques N. M.; Godfrey H. G. W.; Argent S. P.; McPherson L. J. M.; Teat S. J.; Cheng Y.; Frogley M. D.; Cinque G.; Day S. J.; Tang C. C.; Easun T. L.; Rudić S.; Ramirez-Cuesta A. J.; Yang S.; Schröder M. Reversible Coordinative Binding and Separation of Sulfur Dioxide in a Robust Metal–Organic Framework with Open Copper Sites. Nat. Mater. 2019, 18 (12), 1358–1365. 10.1038/s41563-019-0495-0. 31611671

[ref49] Martínez-Ahumada E.; Díaz-Ramírez M. L.; Lara-García H. A.; Williams D. R.; Martis V.; Jancik V.; Lima E.; Ibarra I. A. High and Reversible SO_2_ Capture by a Chemically Stable Cr(III)-Based MOF. J. Mater. Chem. A 2020, 8 (23), 11515–11520. 10.1039/C9TA13524C.

[ref50] Brandt P.; Xing S.-H.; Liang J.; Kurt G.; Nuhnen A.; Weingart O.; Janiak C. Zirconium and Aluminum MOFs for Low-Pressure SO_2_ Adsorption and Potential Separation: Elucidating the Effect of Small Pores and NH_2_ Groups. ACS Appl. Mater. Interfaces 2021, 13 (24), 29137–29149. 10.1021/acsami.1c06003. 34115467

[ref51] Brandt P.; Nuhnen A.; Öztürk S.; Kurt G.; Liang J.; Janiak C. Comparative Evaluation of Different MOF and Non-MOF Porous Materials for SO_2_ Adsorption and Separation Showing the Importance of Small Pore Diameters for Low-Pressure Uptake. Adv. Sustainable Syst. 2021, 5 (4), 2000285 10.1002/adsu.202000285.

[ref52] Chen F.; Lai D.; Guo L.; Wang J.; Zhang P.; Wu K.; Zhang Z.; Yang Q.; Yang Y.; Chen B.; Ren Q.; Bao Z. Deep Desulfurization with Record SO_2_ Adsorption on the Metal–Organic Frameworks. J. Am. Chem. Soc. 2021, 143 (24), 9040–9047. 10.1021/jacs.1c02176. 34115480

[ref53] Simon C. M.; Smit B.; Haranczyk M. PyIAST: Ideal Adsorbed Solution Theory (IAST) Python Package. Comput. Phys. Commun. 2016, 200, 364–380. 10.1016/j.cpc.2015.11.016.

[ref54] Guo Z.; Li Y.; Zhang P.; Cui J.; Chen L.; Yang L.; Wang J.; Cui X.; Xing H. Pillared-Layer Ultramicroporous Material for Highly Selective SO_2_ Capture from CO_2_ Mixtures. Sep. Purif. Technol. 2022, 295, 121337 10.1016/j.seppur.2022.121337.

[ref55] Li W.; Cheng C.; Gao G.; Xu H.; Huang W.; Qu Z.; Yan N. Trace SO_2_ Capture within the Engineered Pore Space Using a Highly Stable SnF_6_^2–^ -Pillared MOF. Mater. Horiz. 2024, 11 (8), 1889–1898. 10.1039/D3MH02222F. 38372122

[ref56] Xing S.; Liang J.; Brandt P.; Schäfer F.; Nuhnen A.; Heinen T.; Boldog I.; Möllmer J.; Lange M.; Weingart O.; Janiak C. Capture and Separation of SO_2_ Traces in Metal–Organic Frameworks via Pre-Synthetic Pore Environment Tailoring by Methyl Groups. Angew. Chem., Int. Ed. 2021, 60 (33), 17998–18005. 10.1002/anie.202105229. PMC8457122 34129750

[ref57] Li L.; da Silva I.; Kolokolov D. I.; Han X.; Li J.; Smith G.; Cheng Y.; Daemen L. L.; Morris C. G.; Godfrey H. G. W.; Jacques N. M.; Zhang X.; Manuel P.; Frogley M. D.; Murray C. A.; Ramirez-Cuesta A. J.; Cinque G.; Tang C. C.; Stepanov A. G.; Yang S.; Schroder M. Post-Synthetic Modulation of the Charge Distribution in a Metal–Organic Framework for Optimal Binding of Carbon Dioxide and Sulfur Dioxide. Chem. Sci. 2019, 10 (5), 1472–1482. 10.1039/C8SC01959B. 30842819 PMC6369579

[ref58] Li J.; Zhou Z.; Han X.; Zhang X.; Yan Y.; Li W.; Smith G. L.; Cheng Y.; MCormick MPherson L. J.; Teat S. J.; Frogley M. D.; Rudić S.; Ramirez-Cuesta A. J.; Blake A. J.; Sun J.; Schröder M.; Yang S. Guest-Controlled Incommensurate Modulation in a Meta-Rigid Metal–Organic Framework Material. J. Am. Chem. Soc. 2020, 142 (45), 19189–19197. 10.1021/jacs.0c08794. 33124803 PMC7668537

[ref59] Cui X.; Yang Q.; Yang L.; Krishna R.; Zhang Z.; Bao Z.; Wu H.; Ren Q.; Zhou W.; Chen B.; Xing H. Ultrahigh and Selective SO_2_ Uptake in Inorganic Anion-Pillared Hybrid Porous Materials. Adv. Mater. 2017, 29 (28), 1606929 10.1002/adma.201606929. 28561930

[ref60] Gong W.; Xie Y.; Yamano A.; Ito S.; Tang X.; Reinheimer E. W.; Malliakas C. D.; Dong J.; Cui Y.; Farha O. K. Modulator-Dependent Dynamics Synergistically Enabled Record SO_2_ Uptake in Zr(IV) Metal–Organic Frameworks Based on Pyrene-Cored Molecular Quadripod Ligand. J. Am. Chem. Soc. 2023, 145 (49), 26890–26899. 10.1021/jacs.3c09648. 38037882

[ref61] Xiong X.-H.; Wei Z.-W.; Wang W.; Meng L.-L.; Su C.-Y. Scalable and Depurative Zirconium Metal–Organic Framework for Deep Flue-Gas Desulfurization and SO_2_ Recovery. J. Am. Chem. Soc. 2023, 145 (26), 14354–14364. 10.1021/jacs.3c03309. 37348117

