# Supplementary material for: Chemically Modified HKUST-1(Cu) for Gas Adsorption and Separation: Mixed-Metal and Hierarchical Porosity
Source: ACS Appl Mater Interfaces. 2024 Nov 12;16(47):65581–91. doi: 10.1021/acsami.4c15059 (PMC11615856; doi:10.1021/acsami.4c15059)
Supplement: Supplementary file 1 — am4c15059_si_001.pdf [file am4c15059_si_001.pdf]

Supporting Information

Chemically Modified HKUST-1(Cu) for Gas Adsorption and Separation: mixed-metal and hierarchical porosity

Ana Yañez-Aulestia*[a], Víctor M. Trejos[a], J. Marcos Esparza-Schulz[a], Ilich A. Ibarra[b,c], and Elí Sánchez-González*[b]

[a] *Laboratorio de Fisicoquímica de Superficies, Departamento de Química, Universidad Autónoma Metropolitana-Iztapalapa (UAM-I), C.P. 09310, Ciudad de México, Mexico.*

[b] *Laboratorio de Fisicoquímica y Reactividad de Superficies (LaFReS), Instituto de Investigaciones en Materiales, Universidad Nacional Autónoma de México, 04510, Ciudad de México, Mexico.*

[c] *On sabbatical as "Catedra Dr Douglas Hugh Everett" at Departamento de Química, Universidad Autónoma Metropolitana-Iztapalapa, San Rafael Atlixco 186, Col. Leyes de Reforma 1ra Seccion, Iztapalapa, C.P. 09310, Ciudad de México, Mexico.*

Corresponding authors' email:

Ana Yañez-Aulestia: anitadelcarmen14@gmail.com

Elí Sánchez-González: elisg@materiales.unam.mx

Content

page

1. Theoretical calculations………………………………………………………... S3
2. Calculations using BETSI Software……………………………………………. S8
3. EDS elemental analyses……………………..………..……..…..……………… S16
4. CO_2 and SO_2 isotherms ant their characterisations……………………………S18
5. Calculation of IAST selectivity…………………………………………..…..………S20

Figures

page

Figure S1. CO_2 adsorption-desorption isotherms at 283, 288, 293 and 298 K for bulk and hierarchical HKUST- materials……………………………………………………… S18
Figure S2. Adsorption-desorption isotherms at 298 K for bulk and hierarchical HKUST-materials for a) CO_2 and b) SO_2 gases…………………………………………….…. S19
Figure S3. The recharacterisation of bulk and hierarchical HKUST-1 materials after gas adsorption. a) FTIR spectra after CO_2, b) FTIR spectra after SO_2 adsorption experiments, c) PXRD after CO_2 and d) PXRD after SO_2 adsorption experiments……………………… S19
Figure S4. Comparison of adsorption isotherms SO_2 (squares), CO_2 (circles), and N_2 (triangles) at 298 K of the bulk and hierarchical materials: a) BH-Cu_{100} vs HH-Cu_{100}, b) BH-$Cu_{90}Ni_{10}$ vs HH-$Cu_{90}Ni_{10}$, c) BH-$Cu_{90}Co_{10}$ vs HH-$Cu_{90}Co_{10}$, and d) BH-$Cu_{90}Rh_{10}$ vs HH-$Cu_{90}Rh_{10}$…………………………………………………………………………….... S21
Figure S5. Langmuir and Dual-Site Langmuir fits of the SO_2 and CO_2 adsorption isotherms of a) BH-Cu_{100}, b) HH-Cu_{100}, c) BH-$Cu_{90}Ni_{10}$, d) HH-$Cu_{90}Ni_{10}$, e) BH-$Cu_{90}Co_{10}$, f) HH-$Cu_{90}Co_{10}$, g) BH-$Cu_{90}Rh_{10}$ and h) HH-$Cu_{90}Rh_{10}$ at 298 K……………………………… S22

1. Theoretical calculations

a) Adsorption model

In this section, we present the statistical associating fluid theory for potentials of variable range (SAFT-VR) to describe the adsorption of chain fluids using a two-dimensional approach. We consider a model of a single-component fluid composed of N spherical particles with a diameter σ in the presence of a uniform wall. The behavior of the particles varies depending on their distance from the wall. The interaction potential exerted by the wall on a particle is denoted as $u_{pw}(z)$, which we can assume to be a function of the perpendicular distance from the wall. In this case, the particle-wall potential is given by,

$$u_{pw}(z) = \begin{cases} \infty, & if \quad z \leq \sigma \\ -\varepsilon_w, & if \quad 0 < z \leq \lambda_w\sigma, \\ 0, & if \quad z > \lambda_w\sigma \end{cases} \tag{1}$$

where z is the perpendicular distance of the particles from the wall, ε_w is the depth, and $\lambda_w\sigma$ is the range of the attractive potential. In our approximation, we describe the system as being composed of two subsystems: a fluid whose particles are near the wall ($z \leq \lambda_w\sigma$), referred to as the "*adsorbed fluid*," and a fluid whose particles are far from the wall ($z > \lambda_w\sigma$), referred to as the "*bulk fluid*." The length scale that characterizes the adsorbed fluid is given by $\lambda_w\sigma$. This approach is valid when the interface between the adsorbed and bulk fluids is not considered. The presence of the wall modifies the properties of the adsorbed and bulk fluids due to the interaction between the molecules and the wall. Therefore, the pair interaction between particles is different for the adsorbed and bulk phases. In the adsorption model, we denote $u_{pp}(r,\varepsilon,\lambda)$ and $u_{pp}^{ads}(r,\varepsilon_{ads},\lambda_{ads})$ as the pair potential for particles in the bulk and adsorbed phases, respectively. Here, ε and λ are the parameters describing the energy depth and range of the potential for the bulk particles, while ε_{ads} and λ_{ads} describe these parameters for the adsorbed particles. In the case of the adsorbed fluid, the pair potential interaction can be described as a decoupling of the x and y coordinates from the z coordinate for each adsorbed particle, as follows,

$$\phi(x,y) = \int dz u_{pp}^{ads}(x,y;\varepsilon_{ads},\lambda_{ads}), \tag{2}$$

where the adsorbed fluid can be approximated by a two-dimensional system, i.e., the pair potential of the adsorbed particles u_{pp}^{ads}, only depends of the coordinates in parallel directions

to the wall. Therefore, the adsorbed fluid can be approximated by a quasi-two dimensional system. The canonical partition function of the adsorbed fluid is given by,

$$Q_{ads}(N,V,T) = \frac{V_{ads}^N}{N!\lambda_B^{3N}} Z_{ads}, \quad (3)$$

where V_{ads} is the adsorbed volume, λ_B, is the de Broglie wavelength, and Z_{ads} is the configurational partition function defined by,

$$Z_{ads} = \frac{1}{V_{ads}^N} \int d^N \vec{r} e^{-\beta U}, \quad (4)$$

where $\beta = \frac{1}{kT}$, and U is the total interaction potential, expressed as the sum of two terms as,

$$U = U_{pw}(z;\sigma,\lambda_w) + U_{pp}^{ads}(x,y), \quad (5)$$

where $U_{pw}(z;\sigma,\lambda_w)$ is the total particle-wall interaction potential ($U_{pw} = N u_{pw}(z)$), and $U_{pp}^{ads}(x,y)$ is the total particles pair potential ($U_{pp}^{ads} = \left(\frac{1}{2}\right) N(N-1)\phi(x,y)$), where the factor $\left(\frac{1}{2}\right) N(N-1)$ corresponds to distinct pairs in the system. In this way, Eq. (4) can be rewritten as,

$$Z_{ads} = \frac{1}{V_{ads}^N} \int d^N z e^{-\beta N u_{pw}(z)} \int d^N x d^N y e^{-\beta \frac{N(N-1)}{2} \phi(x,y)}, \quad (6)$$

where the adsorbed fluid can be characterized by a volume V_{ads}, that corresponds to an adsorption area (S), and perpendicular distance between the wall and the fluid affected by the wall (z_o). Therefore, the Eq. (6) can be rewritten as,

$$Z_{ads} = Z_{1D} Z_{2D}, \quad (7)$$

Where,

$$Z_{1D} = \frac{1}{z_0^N} \int d^N z e^{-\beta N u_{pw}(z)}, \quad (8)$$

$$Z_{2D} = \frac{1}{S^N} \int d^N x d^N y e^{-\beta \frac{N(N-1)}{2} \phi(x,y)}, \quad (9)$$

where Z_{1D} and Z_{2D} are the one and two-dimensional configurational partition functions, respectively. The distance z_o can be written as a function of the range of the attractive potential of the wall and the diameter of the particles ($z_o = \lambda_w \sigma$). In this way, the Eq. (8) can be expressed as,

$$Z_{1D} = \frac{1}{(\lambda_w \sigma)^N} \int d^N z e^{-\beta N u_{pw}(z)} = \left[\frac{1}{\lambda_w \sigma} \int dz\, e^{-\beta u_{pw}(z)}\right]^N \quad (10)$$

The configurational partition function in Eq. (10) can be evaluated using the mean-value theorem, leading to the following expression,

$$Z_{1D} = e^{-\beta N u_{pw}(z')}, \quad (11)$$

where z' is the value of the coordinate z that guarantees the mean value of the Boltzmann factor. The canonical partition function of the adsorbed fluid can be written as,

$$Q_{ads} = Q_{ads}^{1D} Q_{ads}^{2D}, \quad (12)$$

where, $Q_{ads}^{1D} = \frac{z_o^N}{\lambda_B^N} e^{-\beta N u_{pw}(z')}$, (13)

and, $Q_{ads}^{2D} = \frac{S^N}{N! \lambda_B^{2N}} \int dx^N dy^N e^{-\beta \frac{N(N-1)}{2} \phi(x,y)}$, (14)

where rearranging we can obtain,

$$Q_{ads} = Q_{ads}^{2D} \left(\frac{\lambda_w \sigma}{\lambda_B} \right)^N e^{-\beta N u_{pw}(z')}. \quad (15)$$

Applying the standard relation $A_{ads} = -kTln(Q_{ads})$, the Helmholtz free energy of the absorbed fluid is given by,

$$\frac{A_{ads}}{NkT} = \frac{A_{2D}}{NkT} - ln\left(\frac{\lambda_w \sigma}{\lambda_B} \right) + \beta u_{pw}(z'), \quad (16)$$

where A_{2D} is the Helmholtz free energy of a two-dimensional fluid interacting via the potential $\phi(x,y)$, which can be described by perturbation theory using hard-disks as a reference fluid,

$$\frac{A_{2D}}{NKT} = \frac{A_{2D}^{ideal}}{NkT} + \frac{A_{2D}^{mono}}{NkT} + \frac{A_{2D}^{chain}}{NkT}, \quad (17)$$

where A^{ideal} is the ideal free energy, A^{mono} is the excess free energy due to monomer segments, A^{chain} is the contribution due to the formation of the chains of monomers and the subscript *2D* correspond to the 2D-fluid. In this study, the free energy due to intermolecular association is not considered. In the particular case of the wall-particle interaction is given by a square-well interaction of range $\lambda_w \sigma$ and energy depth ε_w, we have that $u_{pw}(z') = -m$ ε_w. On the other hand, we consider an analogous perturbation expression for the bulk fluid,

$$\frac{A_{3D}}{NKT} = \frac{A_{3D}^{ideal}}{NkT} + \frac{A_{3D}^{mono}}{NkT} + \frac{A_{3D}^{chain}}{NkT}, \quad (18)$$

where the Helmholtz free energy is denoted with the subscript 3D to refer to a three-dimensional fluid. Once we have suitable expressions for the Helmholtz free energy in both three and two dimensions, the adsorption isotherms can be determined at a given temperature by solving the chemical equilibrium between the adsorbed and bulk phases as follows,

$$\mu_{ads} = \mu_b, \quad (19)$$

where, $\mu_{ads} = \left(\frac{\partial A_{ads}}{\partial N_{ads}} \right)_{T,V}$, (20)

and, $\mu_b = \left(\frac{\partial A_b}{\partial N_b}\right)_{T,V}$, (21)

where μ_{ads} and μ_{2D} are the chemical potentials for the adsorbed and 3D fluids, respectively, and $A_{3D} \equiv A_b$ and A_{ads} are the Helmholtz free energies for the 3D bulk and adsorbed systems formed by N_b and N_{ads} particles, respectively. It is important to note that the particle-particle interactions are described by the square-well (SW) pair potential. More detailed information about the *3D* and *2D* expressions for the Helmholtz free energy can be found in Refs.[1-7]

b. Adsorption of Carbon Dioxide

The theory presented in the previous section has been extended to the case of carbon dioxide adsorption within the SAFT-VR framework.[1] In this context, we consider carbon dioxide as composed of *N* molecules, each made up of m spherical segments of diameter σ. The particle-particle and particle-wall interactions are assumed to be described by a SW pair potential. The prediction of adsorption isotherms using the SAFT-VR approach is based on solving the thermodynamic equilibrium between the bulk and adsorbed phases, determined by the equality of the chemical potentials, as given by Eq. (19). The chemical potentials are derived from the SAFT-VR expressions for the Helmholtz free energies for 3D and 2D chain-molecule fluids.[1-8] Seven molecular parameters are required to determine the adsorption isotherms for the carbon dioxide system, assuming the diameter of the particles remains unchanged when adsorbed onto a surface. These parameters correspond to three types of SW interactions as follows: *i*) particle-particle interaction for the bulk phase: σ, λ, and ε, *ii*) particle-particle interaction for the adsorbed phase: σ, λ_{ads}, and ε_{ads} and *iii*) Particle-wall interaction: ε_w, λ_w. The parameters used for predicting the adsorption isotherms of carbon dioxide are reported in Tables S1 and S2. Further details and explanations of each parameter can be found in Refs.[1-8]

Table S1. The molecular parameters used to describe carbon dioxide adsorption on different surfaces are detailed below. The parameters σ, λ, and ε, corresponding to the bulk phase.[9]

Particles in the adsorbed phase have the same diameter σ, while the SW attractive parameters (λ_{ads}, ε_{ads}) were obtained following the same procedure used in our previous work.[2-8] The particle-wall SW attractive range is given by λ_w.

Substance	m	λ	$\sigma/\grave{A}$	$(\varepsilon/k)/K$	λ_{ads}	$(\varepsilon_{ads}/k)/$	λ_w
CO_2	2.00	1.5257	2.7864	179.27	1.262	143.416	0.8165

Table S2. Optimised values for the particle-wall energy parameter ε_w for carbon dioxide adsorption onto different substrates. Results are given for the SAFT-VR approach used in this work. Experimental and calculated values of the BET specific surface area (S_{BET}) and calculated isosteric heats are reported at 298 K. The different substrates correspond to different metal-organic frameworks (MOFs).

**Adsorbent**	$S_{BET}^{BETSI}(m^2/g)$	$S_{BET}^{cal}(m^2/g)$	$\varepsilon_w(kJ/mol)$
B-HCu100	1654	1200	6.671
H-HCu100	1666	1800	8.417
B-HCu90Co10	1242	920	6.853
H-HCu90Co10	1723	520	7.448
B-Cu90Ni10	2585	1520	6.510
H-HCu90Ni10	1892	920	6.570
B-HCu90Rh10	873	930	6.808
H-HCu90Rh10	1997	1550	6.332

2. Calculations using BETSI Software[10]

a. BH-Cu_{100}

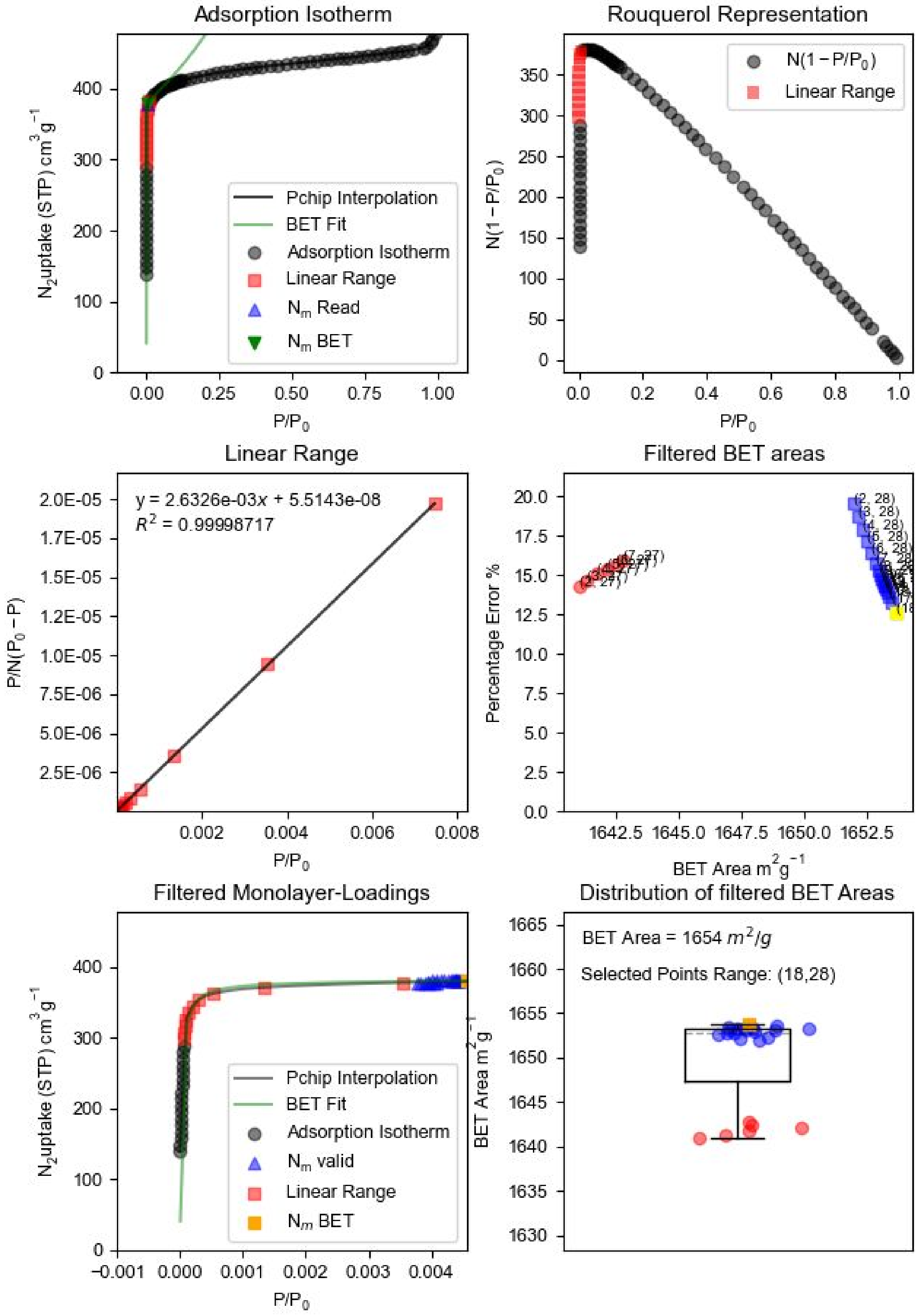

b. HH-Cu_{100}

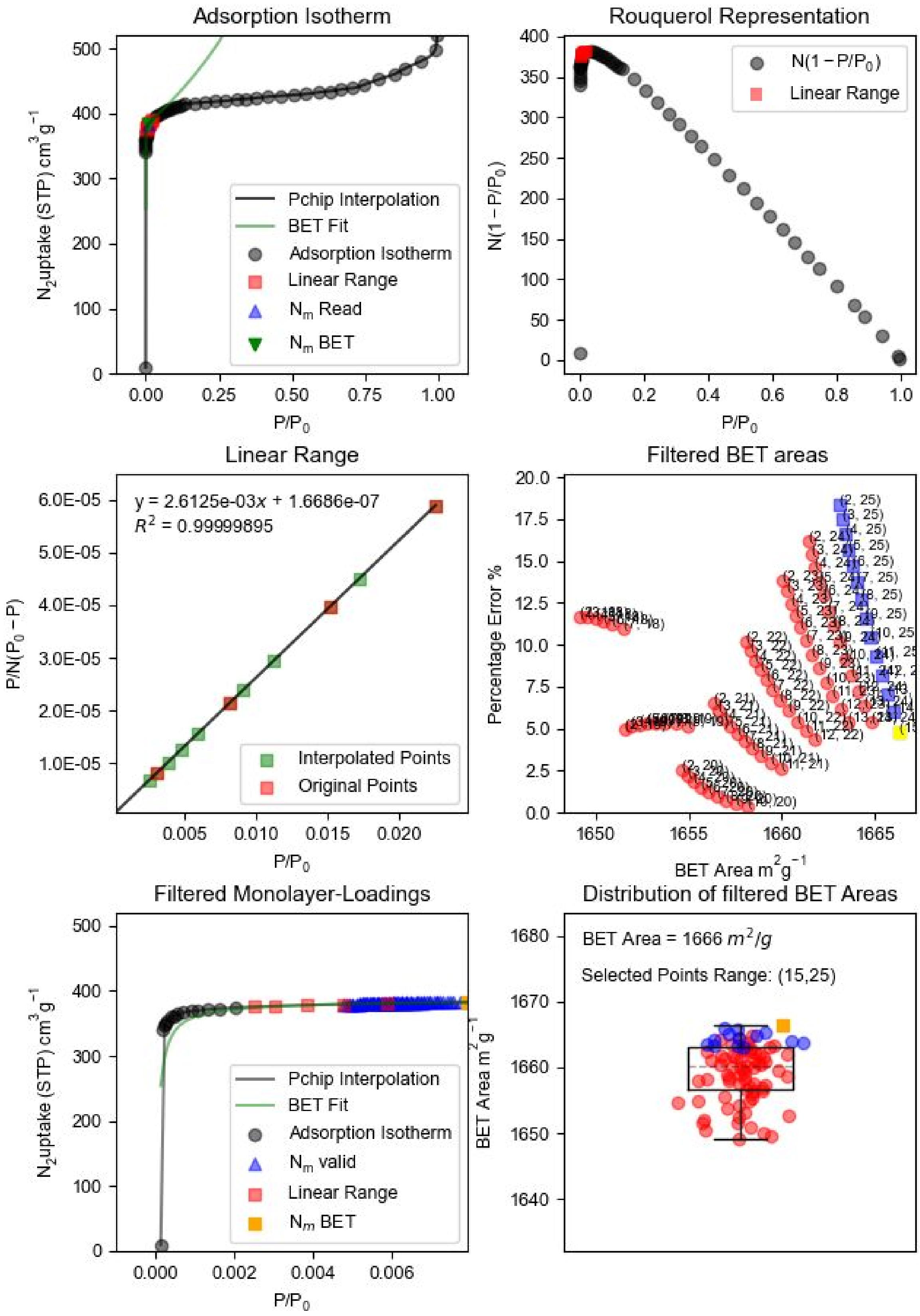
Adsorption Isotherm
Pchip Interpolation
BET Fit
Adsorption Isotherm
Linear Range
N_m Read
N_m BET
N_2 uptake (STP) cm^3 g^-1
P/P_0
Rouquerol Representation
N(1 − P/P_0)
Linear Range
N(1 − P/P_0)
P/P_0
Linear Range
y = 2.6125e-03x + 1.6686e-07
R^2 = 0.99999895
Interpolated Points
Original Points
P/N(P_0 − P)
P/P_0
Filtered BET areas
Percentage Error %
BET Area m^2 g^-1
Filtered Monolayer-Loadings
Pchip Interpolation
BET Fit
Adsorption Isotherm
N_m valid
Linear Range
N_m BET
N_2 uptake (STP) cm^3 g^-1
P/P_0
Distribution of filtered BET Areas
BET Area = 1666 m^2/g
Selected Points Range: (15,25)
BET Area m^2 g^-1

c. BH-$Cu_{90}Ni_{10}$

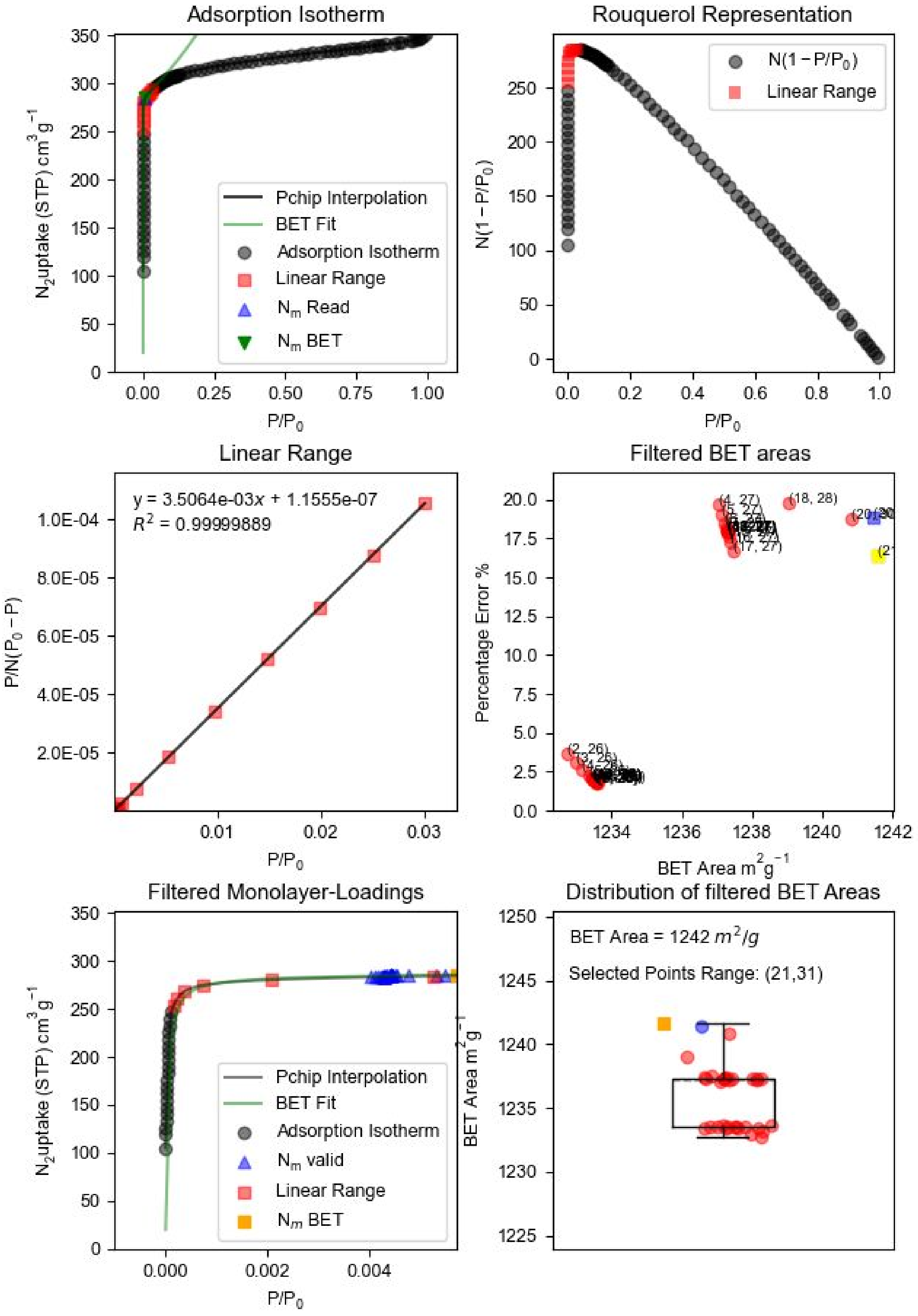

d. HH-$Cu_{90}Ni_{10}$

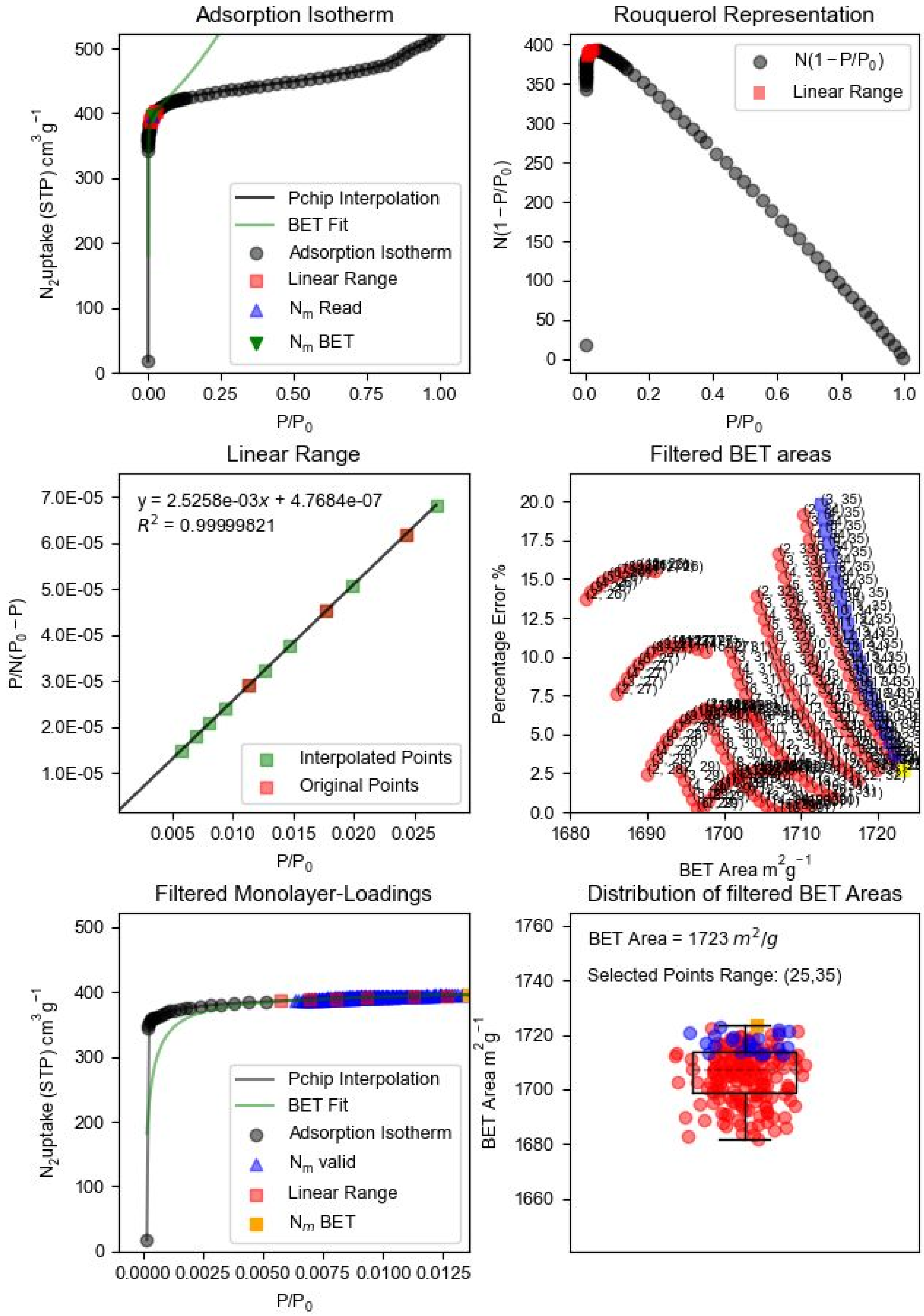

e. BH-$Cu_{90}Co_{10}$

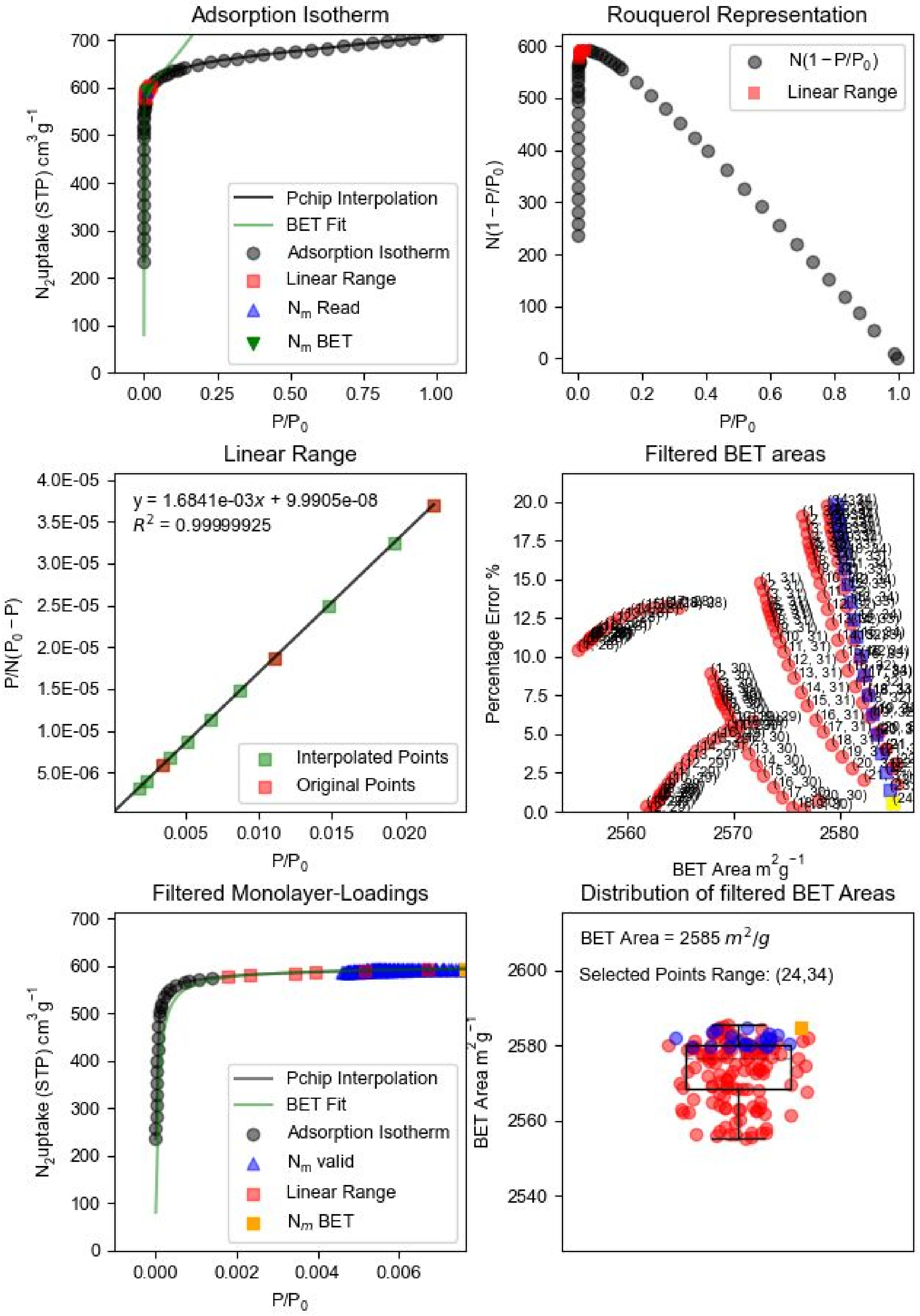

f. HH-$Cu_{90}Co_{10}$

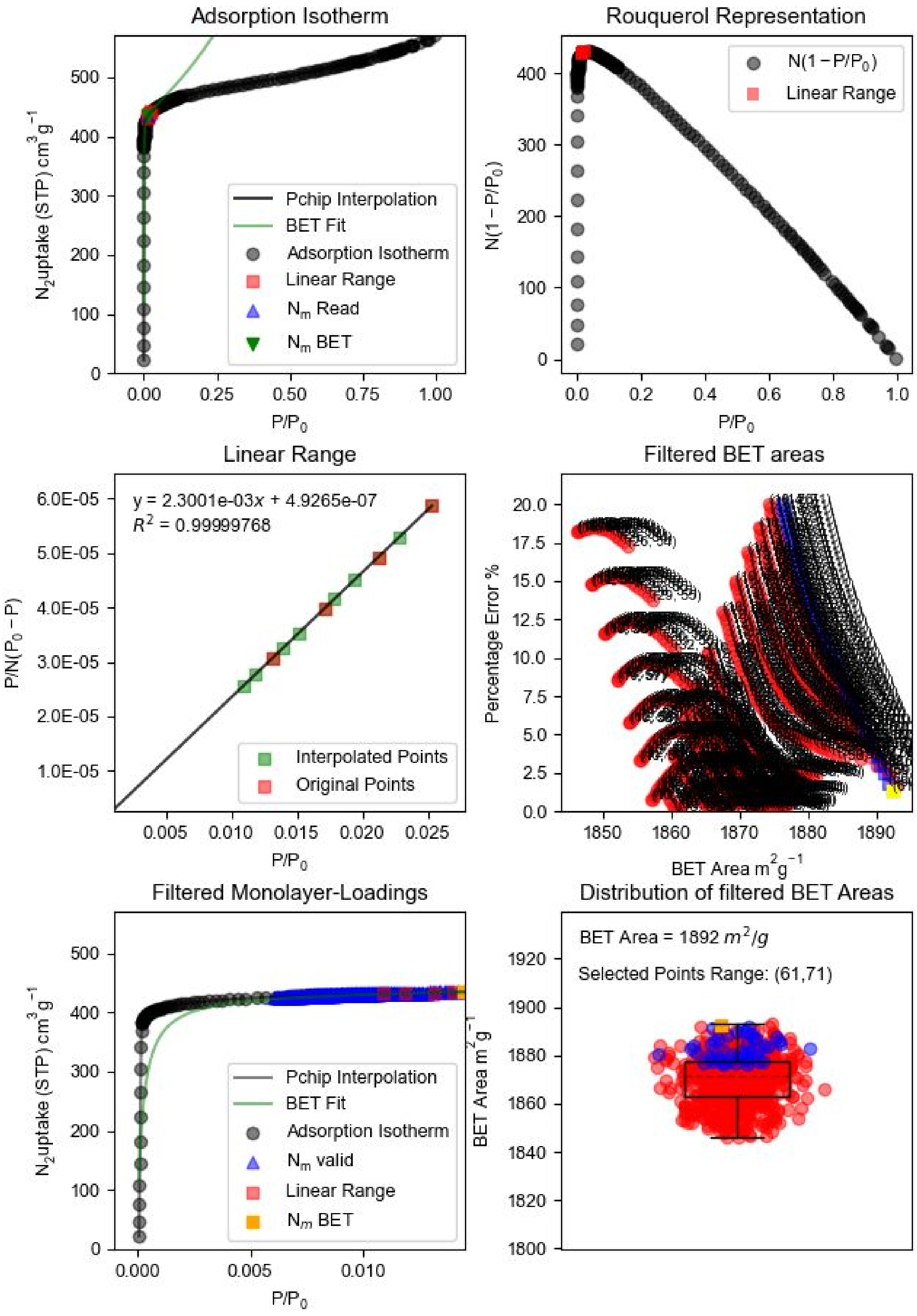

g. BH-$Cu_{90}Rh_{10}$

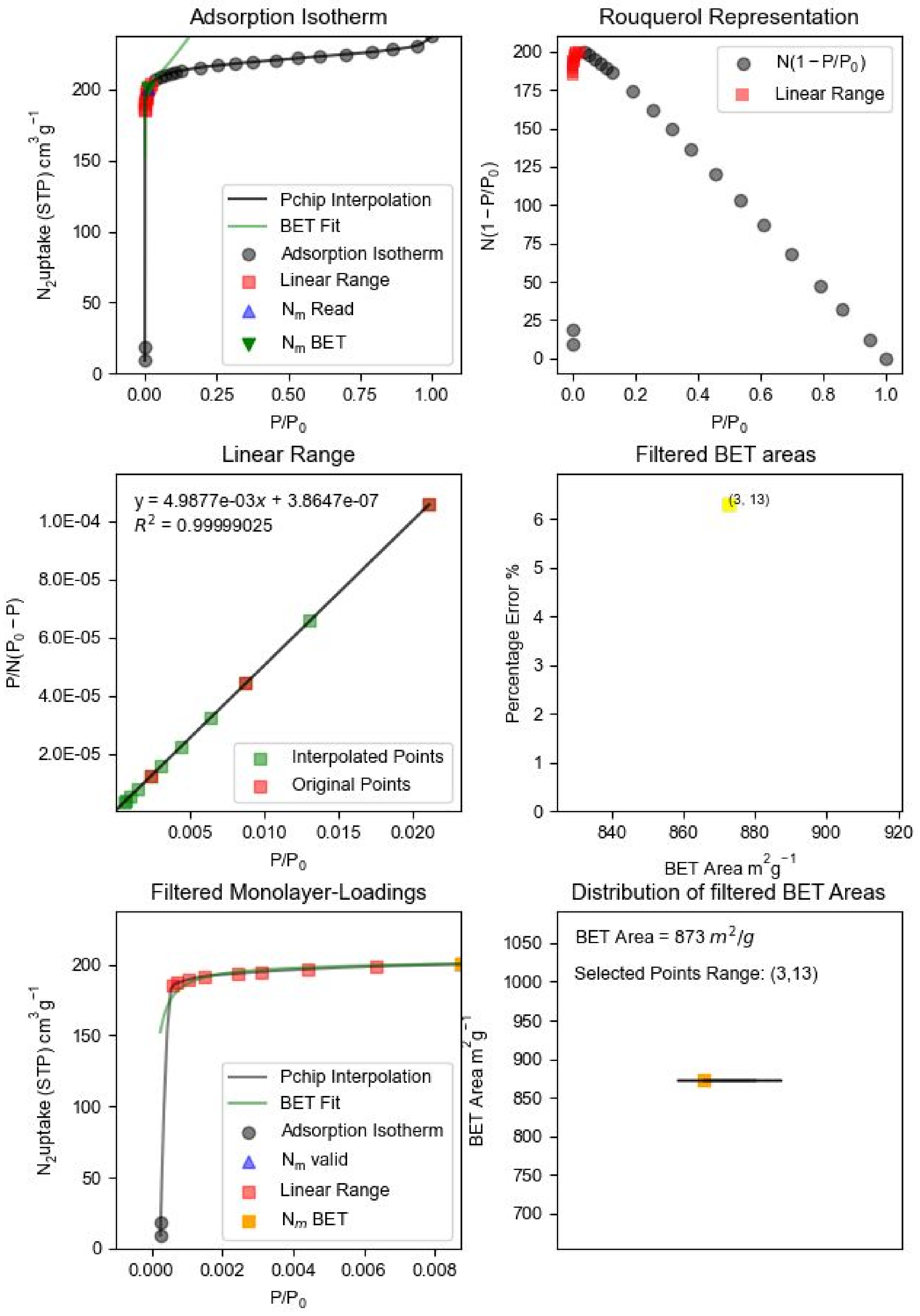

h. HH-$Cu_{90}Rh_{10}$

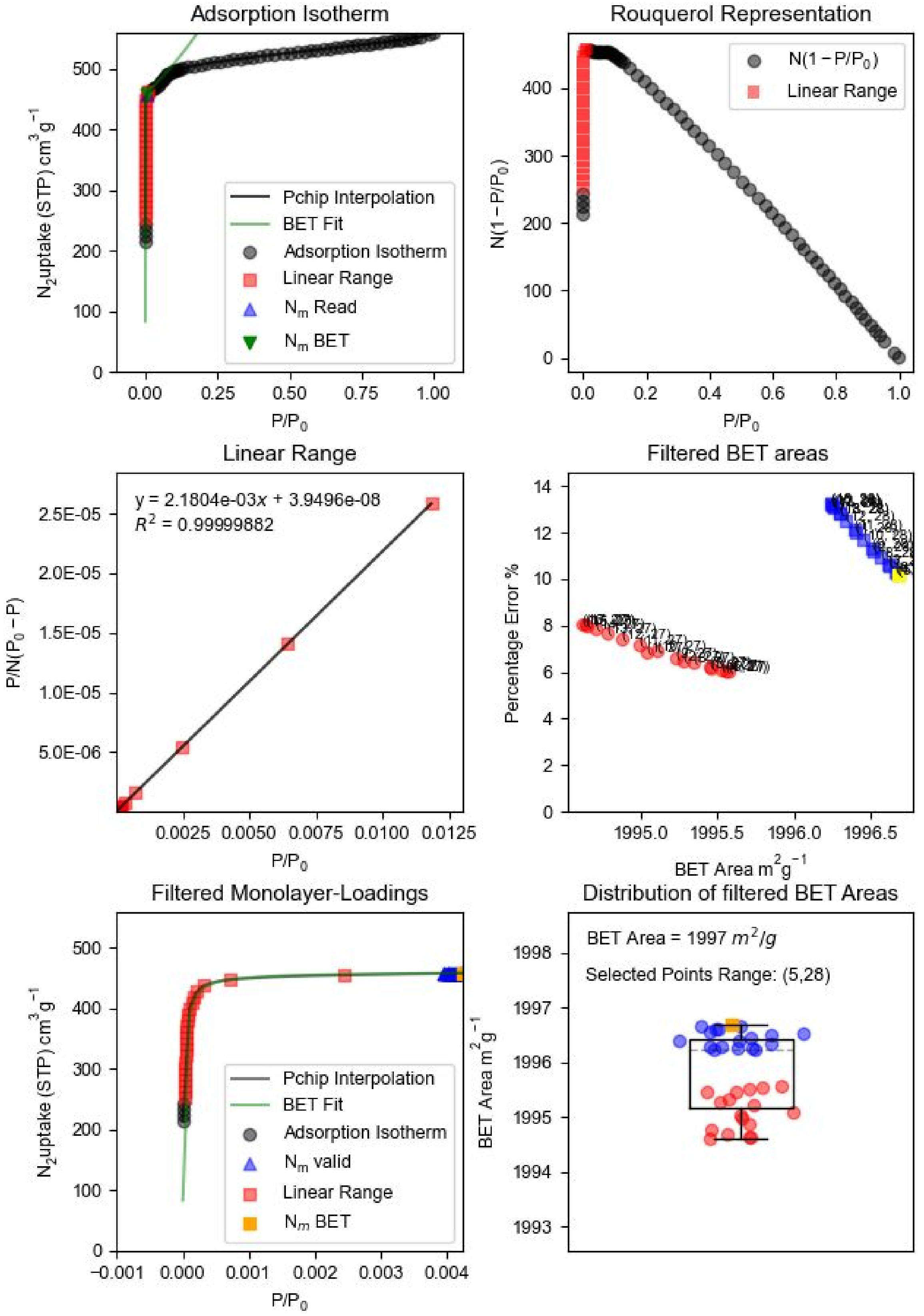

3. EDS elemental analyses

Table S3. Elemental composition of HKUST-1 bulk and hierarchical samples.

Material	weight%				mol		mol%
	C	O	Cu	Me	Cu	Me	Cu:Me
BH-Cu_{100}	33.99	36.91	29.10	0.00	0.458	-	100:0
HH-Cu_{100}	32.61	38.71	28.68	0.00	0.451	-	100:0
BH-$Cu_{90}Ni_{10}$	34.53	36.18	27.18	2.11	0.428	0.036	92.2:7.8
HH-$Cu_{90}Ni_{10}$	33.65	35.47	28.75	2.13	0.452	0.036	92.6:7.4
BH-$Cu_{90}Co_{10}$	34.42	36.22	27.13	2.23	0.427	0.038	91.9:8.1
HH-$Cu_{90}Co_{10}$	34.34	35.67	27.83	2.16	0.438	0.037	92.3:7.7
BH-$Cu_{90}Rh_{10}$	31.68	34.51	29.43	4.38	0.463	0.043	91.6:8.4
HH-$Cu_{90}Rh_{10}$	31.86	35.62	28.75	3.77	0.452	0.037	92.5:7.5

BH-Cu_{100}

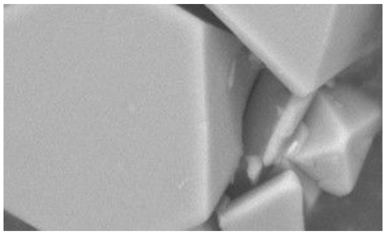

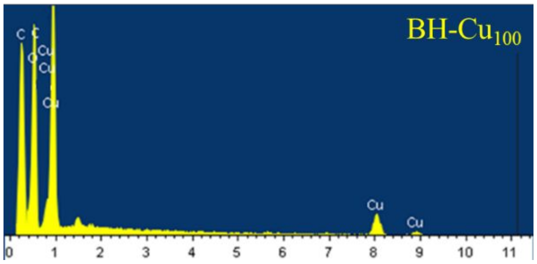

HH-Cu_{100}

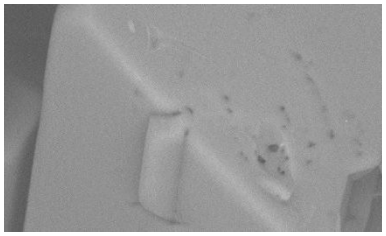

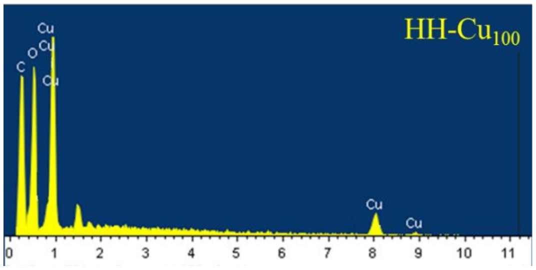

BH-$Cu_{90}Ni_{10}$

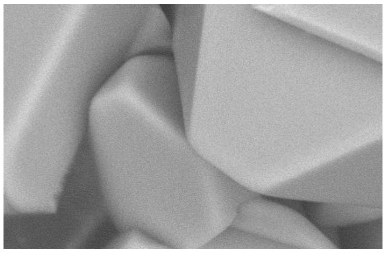

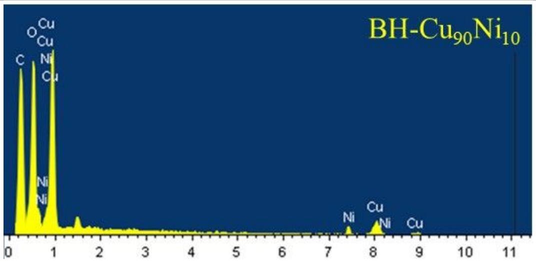

**HH-$Cu_{90}Ni_{10}$**	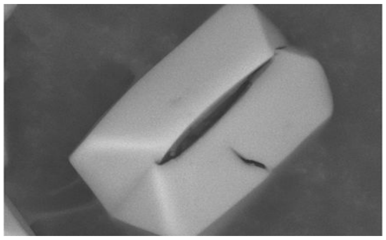	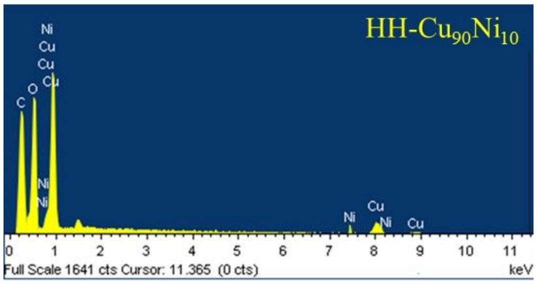
**BH-$Cu_{90}Co_{10}$**	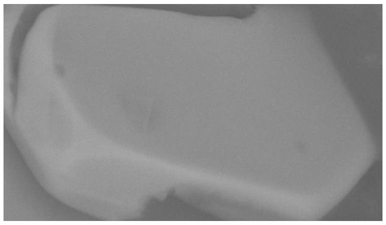	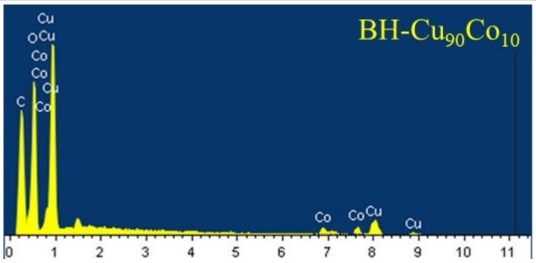
**HH-$Cu_{90}Co_{10}$**	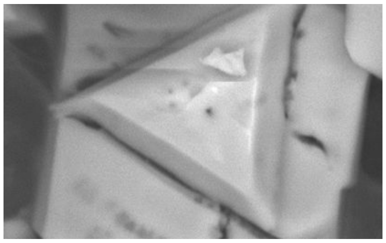	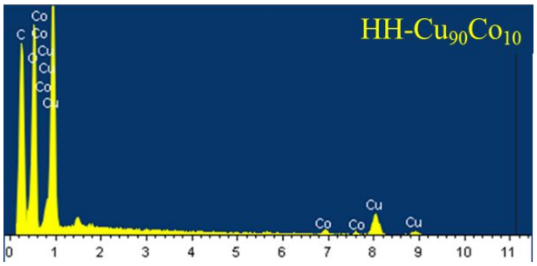
**BH-$Cu_{90}Rh_{10}$**	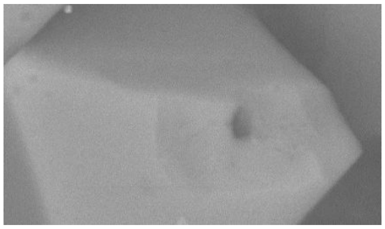	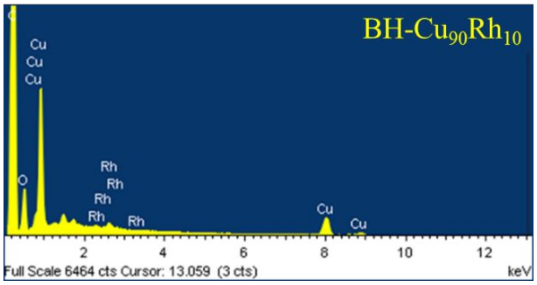
**HH-$Cu_{90}Rh_{10}$**	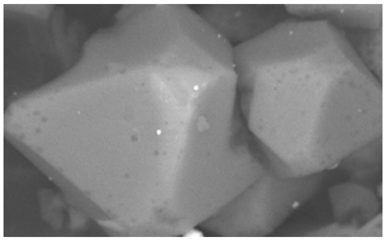	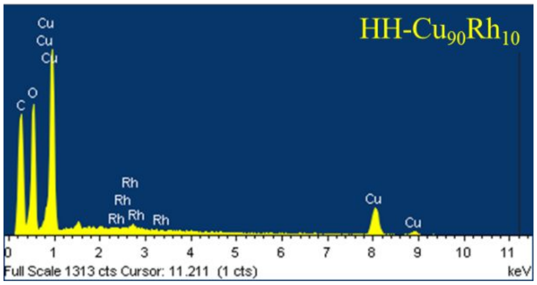

4. CO_2 and SO_2 isotherms ant their characterisations

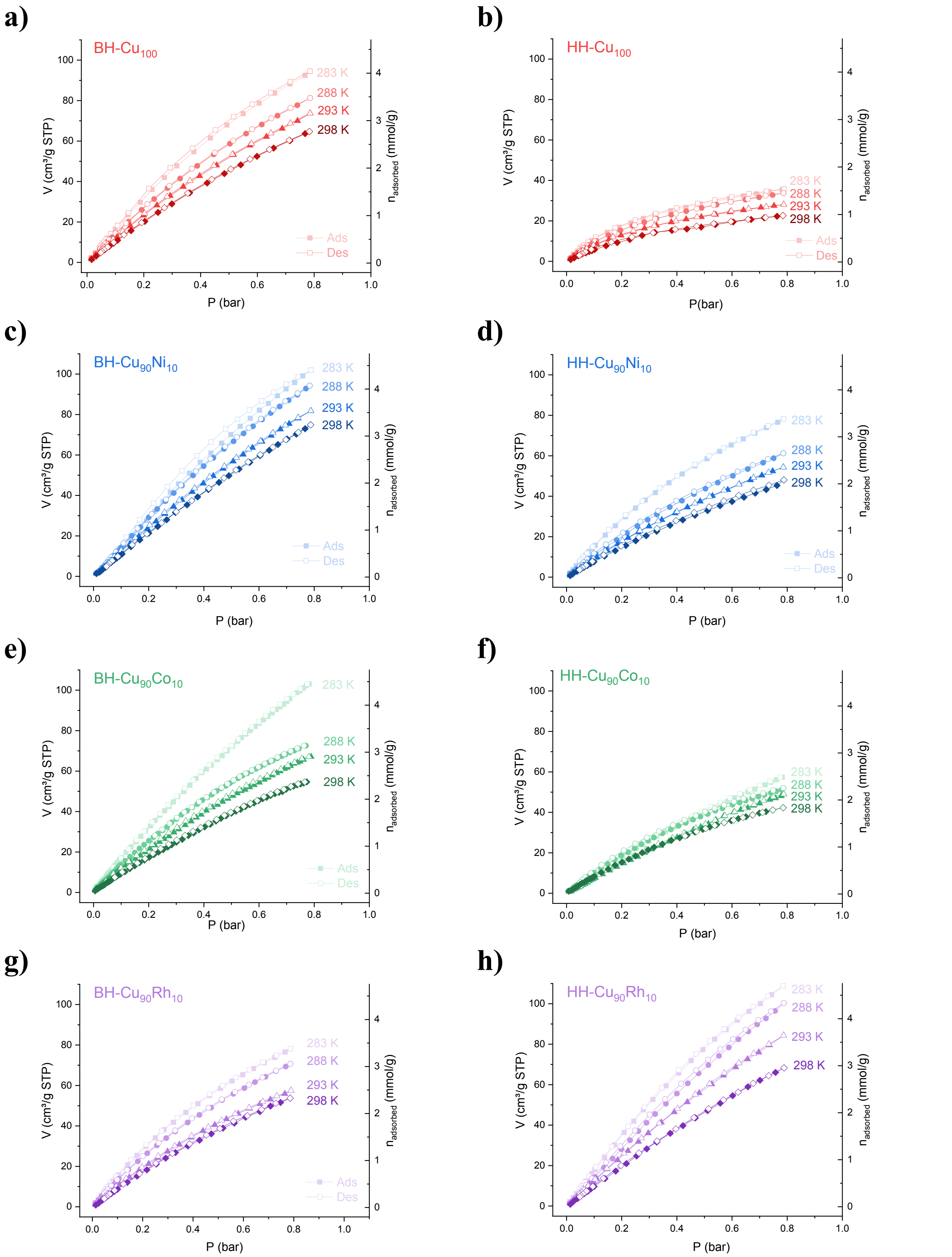

Figure S1. CO_2 adsorption-desorption isotherms at 283, 288, 293 and 298 K for bulk and hierarchical HKUST- materials.

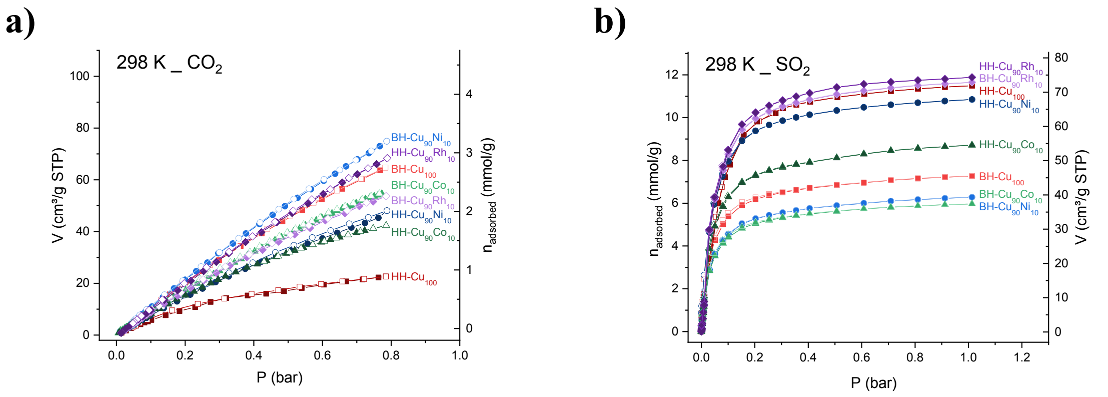

Figure S2. Adsorption-desorption isotherms at 298 K for bulk and hierarchical HKUST-materials for a) CO_2 and b) SO_2 gases.

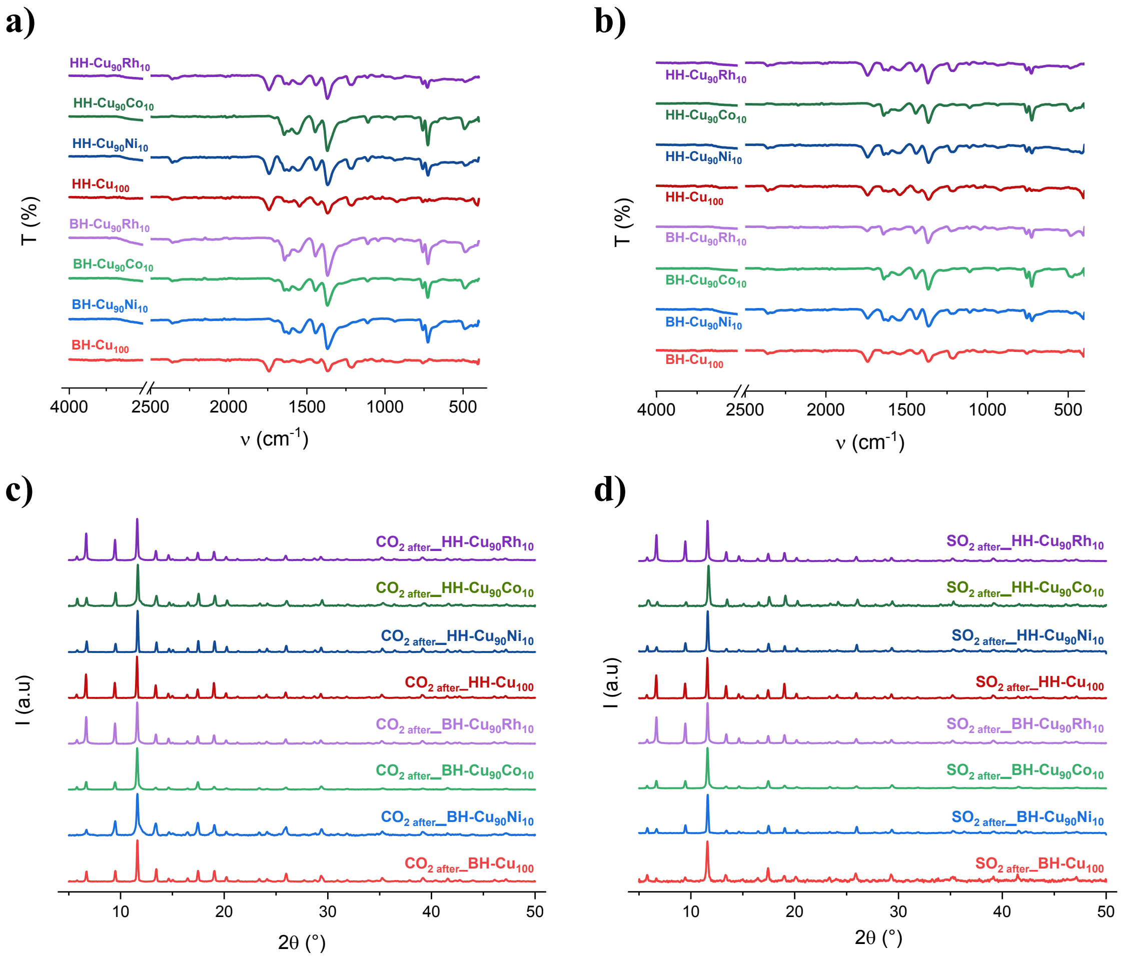

Figure S3. The recharacterisation of bulk and hierarchical HKUST-1 materials after gas adsorption. a) FTIR spectra after CO_2, b) FTIR spectra after SO_2 adsorption experiments, c) PXRD after CO_2 and d) PXRD after SO_2 adsorption experiments.

5. Calculation of IAST selectivity

The acid gas mixture separation potential of the materials was assessed using the Ideal Adsorbed Solution Theory (IAST). The binary mixture SO_2/CO_2 selectivity was estimated using the pyIAST package. [10] First, the adsorption branch for the single-component isotherms of SO_2 and CO_2 collected at 298 K were fitted using the Langmuir (22) or dual-site Langmuir (23) equations.

$$n = \mathrm{M}\frac{Kp}{1+Kp} \tag{22}$$

$$n = M_1\frac{K_1p}{1+K_1p} + M_2\frac{K_2p}{1+K_2p} \tag{23}$$

IAST selectivity was calculated using the following formula:

$$S = \frac{q_1/q_2}{y_1/y_2} \tag{24}$$

where q_i is the adsorption loading and y_i is the molar fraction of each gas.

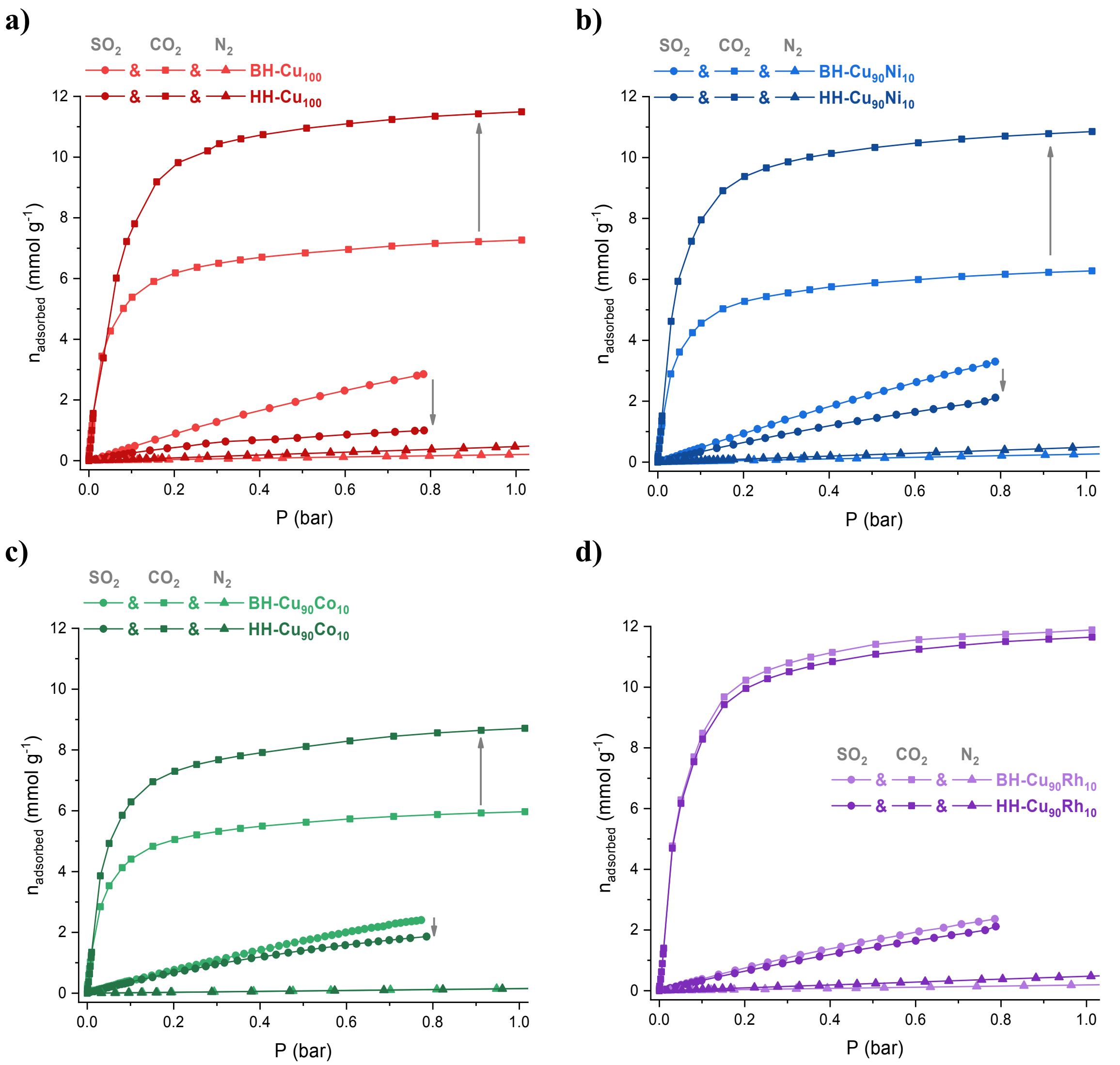

Figure S4. Comparison of adsorption isotherms SO_2 (squares), CO_2 (circles), and N_2 (triangles) at 298 K of the bulk and hierarchical materials: a) BH-Cu_{100} vs HH-Cu_{100}, b) BH-$Cu_{90}Ni_{10}$ vs HH-$Cu_{90}Ni_{10}$, c) BH-$Cu_{90}Co_{10}$ vs HH-$Cu_{90}Co_{10}$, and d) BH-$Cu_{90}Rh_{10}$ vs HH-$Cu_{90}Rh_{10}$.

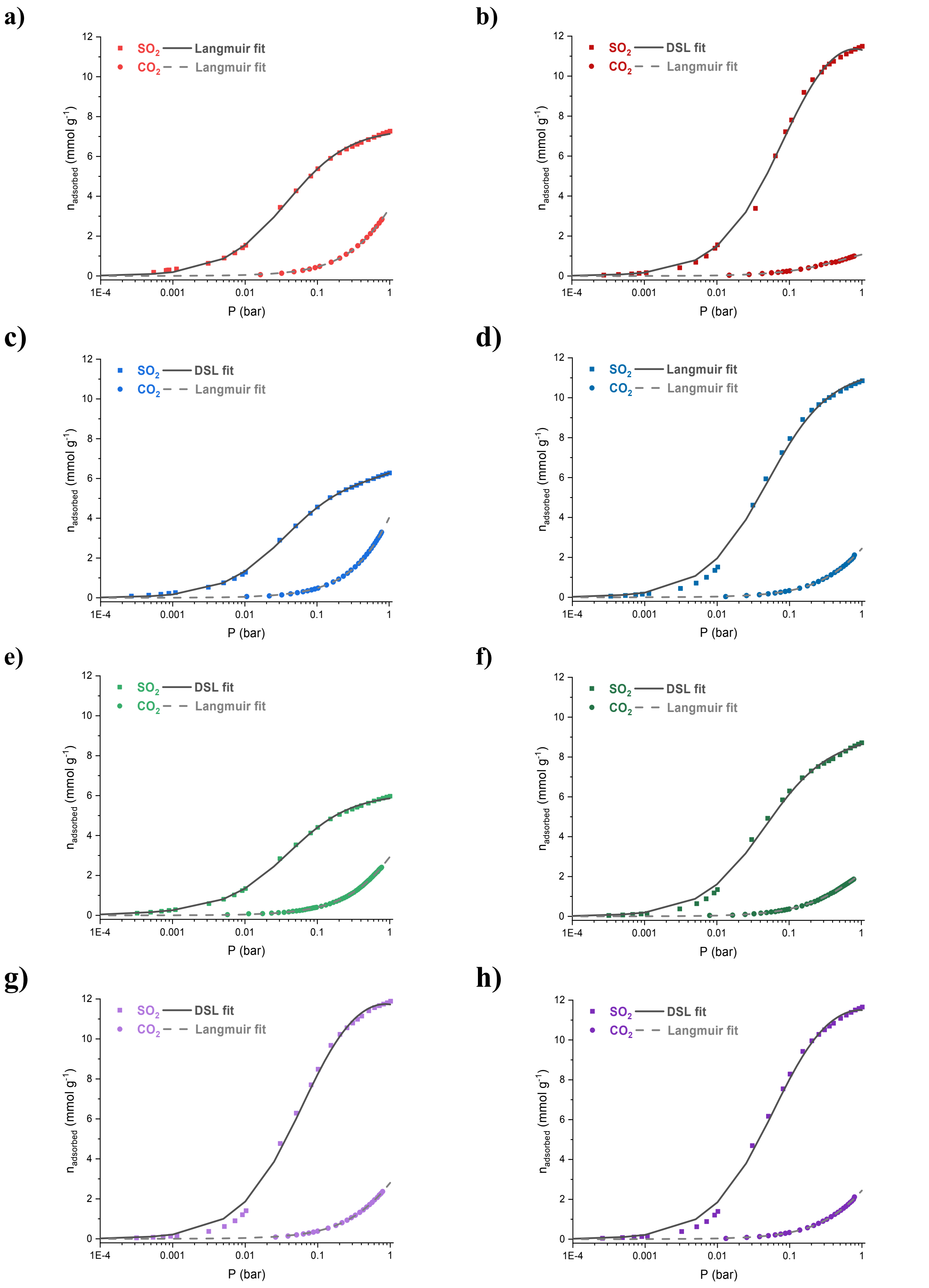

Figure S5. Langmuir and Dual-Site Langmuir fits of the SO_2 and CO_2 adsorption isotherms of a) BH-Cu_{100}, b) HH-Cu_{100}, c) BH-$Cu_{90}Ni_{10}$, d) HH-$Cu_{90}Ni_{10}$, e) BH-$Cu_{90}Co_{10}$, f) HH-$Cu_{90}Co_{10}$, g) BH-$Cu_{90}Rh_{10}$ and h) HH-$Cu_{90}Rh_{10}$ at 298 K.

References

(1) A. Gil-Villegas, A. Galindo, P. J. Whitehead, S. J. Mills, G. Jackson, and A. N. Burgess, "Statistical associating fluid theory for chain molecules with attractive potentials of variable range," *J. Chem. Phys.* **1997***,* 106, 4168-4186.

(2) A. Martínez, M. Castro, C. McCabe, and A. Gil-Villegas, "Predicting adsorption isotherms using a two-dimensional statistical associating fluid theory," *J. Chem. Phys.* **2007**, 126, 074707(1)-074707(7).

(3) G. Jiménez-Serratos, S. Santillán, C. Avendaño, M. Castro, and A. Gil-Villegas, "Molecular thermodynamics of adsorption using discrete-potential systems," O*il Gas Sci. Technol.* **2008**, 63, 329-341.

(4) M. Castro, J. M. de la Cruz, E. Buenrostro-González, S. Lopéz-Ramírez, and A. Gil-Villegas, "Predicting adsorption isotherms of asphaltenes in porous materials," *Fluid Phase Equilib*. **2009**, 87,113-119.

(5) M. Castro, A. Martínez, and A. Gil-Villegas, "Modelling adsorption isotherms of binary mixtures of carbon dioxide, methane and nitrogen," *Adsorpt. Sci. Technol.* **2011**, 29 (1), 59-70.

(6) V. M. Trejos, M. Becerra, S. Figueroa-Gerstenmaier, and A. Gil-Villegas, "Theoretical modelling of adsorption of hydrogen onto graphene, mofs and other carbon-based substrates," *Mol. Phys.* **2014**, 112 (17), 2330-2338.

(7) A. Martínez, V. M. Trejos, A. Gil-Villegas, "Predicting adsorption isotherms for methanol and water onto different surfaces using the SAFT-VR-2D approach and molecular simulation," *Fluid Phase Equilib.* **2017**, 449, 207-216.

(8) V. M. Trejos, A. Martínez, and A. Gil-Villegas, "Semiclassical SAFT-VR-2D modeling of adsorption selectivities for binary mixtures of hydrogen and methane adsorbed onto MOFs," *Fluid Phase Equilib*. **2018**, 462, 153-171.

(9) C. M. Colina, A. Galindo, F. J. Blas, K. E. Gubbins, "Phase behavior of carbon dioxide mixtures with n-alkanes and *n*-perfluoroalkanes," *Fluid Phase Equilib.* **2004**, 222, 77-85.

(10) Osterrieth, J. W. M.; Rampersad, J.; Madden, D.; Rampal, N.; Skoric, L.; Connolly, B.; Allendorf, M. D.; Stavila, V.; Snider, J. L.; Ameloot, R.; Marreiros, J.; Ania, C.; Azevedo, D.; Vilarrasa-Garcia, E.; Santos, B. F.; Bu, X. H.; Chang, Z.; Bunzen, H.; Champness, N. R.;

Griffin, S. L.; Chen, B.; Lin, R. B.; Coasne, B.; Cohen, S.; Moreton, J. C.; Colón, Y. J.; Chen, L.; Clowes, R.; Coudert, F. X.; Cui, Y.; Hou, B.; D'Alessandro, D. M.; Doheny, P. W.; Dincă, M.; Sun, C.; Doonan, C.; Huxley, M. T.; Evans, J. D.; Falcaro, P.; Ricco, R.; Farha, O.; Idrees, K. B.; Islamoglu, T.; Feng, P.; Yang, H.; Forgan, R. S.; Bara, D.; Furukawa, S.; Sanchez, E.; Gascon, J.; Telalović, S.; Ghosh, S. K.; Mukherjee, S.; Hill, M. R.; Sadiq, M. M.; Horcajada, P.; Salcedo-Abraira, P.; Kaneko, K.; Kukobat, R.; Kenvin, J.; Keskin, S.; Kitagawa, S.; Otake, K. ichi; Lively, R. P.; DeWitt, S. J. A.; Llewellyn, P.; Lotsch, B. V.; Emmerling, S. T.; Pütz, A. M.; Martí-Gastaldo, C.; Padial, N. M.; García-Martínez, J.; Linares, N.; Maspoch, D.; Suárez del Pino, J. A.; Moghadam, P.; Oktavian, R.; Morris, R. E.; Wheatley, P. S.; Navarro, J.; Petit, C.; Danaci, D.; Rosseinsky, M. J.; Katsoulidis, A. P.; Schröder, M.; Han, X.; Yang, S.; Serre, C.; Mouchaham, G.; Sholl, D. S.; Thyagarajan, R.; Siderius, D.; Snurr, R. Q.; Goncalves, R. B.; Telfer, S.; Lee, S. J.; Ting, V. P.; Rowlandson, J. L.; Uemura, T.; Iiyuka, T.; van der Veen, M. A.; Rega, D.; Van Speybroeck, V.; Rogge, S. M. J.; Lamaire, A.; Walton, K. S.; Bingel, L. W.; Wuttke, S.; Andreo, J.; Yaghi, O.; Zhang, B.; Yavuz, C. T.; Nguyen, T. S.; Zamora, F.; Montoro, C.; Zhou, H.; Kirchon, A.; Fairen-Jimenez, D. How Reproducible Are Surface Areas Calculated from the BET Equation? *Adv. Mater.* **2022**, 34 (27). https://doi.org/10.1002/adma.202201502.

(11) A. Nuhnena and C. Janiak, A practical guide to calculate the isosteric heat/enthalpy of adsorption via adsorption isotherms in metal–organic frameworks, MOFs, *Dalton Trans.* **2020**, 49, 10295-10307. https://doi.org/10.1039/D0DT01784A
